# Paring Down HIV Env: Design and Crystal Structure of a Stabilized Inner Domain of HIV-1 gp120 Displaying a Major ADCC Target of the A32 Region

**DOI:** 10.1016/j.str.2016.03.005

**Published:** 2016-05-03

**Authors:** William D. Tolbert, Neelakshi Gohain, Maxime Veillette, Jean-Philippe Chapleau, Chiara Orlandi, Maria L. Visciano, Maryam Ebadi, Anthony L. DeVico, Timothy R. Fouts, Andrés Finzi, George K. Lewis, Marzena Pazgier

**Affiliations:** 1 Division of Vaccine Research, Institute of Human Virology, University of Maryland School of Medicine, Baltimore, MD 21201, USA; 2 Department of Biochemistry and Molecular Biology, University of Maryland School of Medicine, Baltimore, MD 21201, USA; 3 Centre de Recherche du CHUM, Université de Montréal, Montreal, QC H2X 0A9, Canada; 4 Department of Microbiology, Infectiology and Immunology, Université de Montréal, Montreal, QC H2X 0A9, Canada; 5 Department of Microbiology and Immunology, University of Maryland School of Medicine, Baltimore, MD 21201, USA; 6 Department of Medicine, University of Maryland School of Medicine, Baltimore, MD 21201, USA; 7 Profectus BioSciences, Inc., Baltimore, MD 21224, USA; 8 Department of Microbiology and Immunology, McGill University, Montreal, QC H2X 0A9, Canada

## Abstract

Evidence supports a role of antibody-dependent cellular cytotoxicity (ADCC) toward transitional epitopes in the first and second constant (C1-C2) regions of gp120 (A32-like epitopes) in preventing HIV-1 infection and in vaccine-induced protection. Here, we describe the first successful attempt at isolating the inner domain (ID) of gp120 as an independent molecule that encapsulates the A32-like region within a minimal structural unit of the HIV-1 Env. Through structure-based design, we developed ID2, which consists of the ID expressed independently of the outer domain and stabilized in the CD4-bound conformation by an inter-layer disulfide bond. ID2 expresses C1-C2 epitopes in the context of CD4-triggered full-length gp120 but without any known neutralizing epitope present. Thus, ID2 represents a novel probe for the analysis and/or selective induction of antibody responses to the A32 epitope region. We also present the crystal structure of ID2 complexed with mAb A32, which defines its epitope.

## Highlights

• ID2 consists of the gp120 inner domain expressed independently of the outer domain
• ID2 displays the A32 ADCC epitope within minimal structural unit of the HIV-1 Env
• ID2 is stabilized in the CD4-bound conformation by an inter-layer disulfide bond
• The A32 epitope maps to layers 1 and 2 of the C1-C2 region of gp120

## Graphical Abstract

**Figure:**
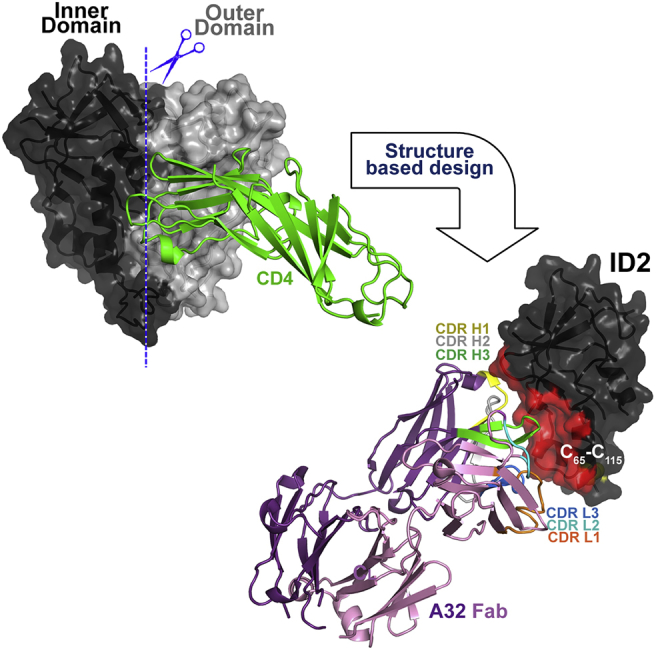

## Teaser

Tolbert at al. describe a novel construct, ID2, consisting of inner domain of gp120 expressed independently of outer domain. ID2 expresses the A32 ADCC epitope within a minimal structural unit of the HIV-1 Env, thus it is a novel probe for the analysis and/or selective induction of A32-like antibody responses.

## Introduction

Numerous studies have indicated a relevant role for Fc receptor (FcR)-effector functions including antibody-dependent cellular cytotoxicity (ADCC) in protective immunity, as these responses have been shown to correlate with slower progression of HIV disease (Jia et al., 2013, Wren et al., 2013) or decreased virus replication. Studies of SIV or SHIV-infected non-human primates, HIV-1-infected humanized mouse models, and the RV144 clinical trial have linked ADCC with post-infection control of viremia and/or the blocking of HIV-1 acquisition, often in the absence of neutralization (Chung et al., 2014, Fouts et al., 2015, Haynes et al., 2012, Rerks-Ngarm et al., 2009, Yates et al., 2014). In particular, the immune correlates analyses of the RV144 trial associated high ADCC activity (together with low immunoglobulin A levels) and polyfunctional immunoglobulin G3 (IgG3) antibodies (Abs) with a reduced risk of infection, thus potentially contributing to vaccine efficacy (Chung et al., 2014, Tomaras et al., 2013, Yates et al., 2014).

Considerable evidence suggests that potent ADCC responses in humans are directed against A32-like epitopes of the Cluster A region (Guan et al., 2013) which map to the first and second constant (C1-C2) region of gp120 (reviewed in Lewis et al., 2014, Pollara et al., 2013, Veillette et al., 2016). These epitopes are classified based on whether they are extant on target cells during viral entry, prior to productive infection of a target cell (entry targets) or exposed on an infected cell producing virions (release targets) (Lewis et al., 2014, Pollara et al., 2013). The A32-like entry targets become exposed during the initial steps of viral entry after Env trimers engage the host CD4 receptor and, possibly, the co-receptor. They persist on newly infected cell surfaces for extended periods of time (Finnegan et al., 2001, Finnegan et al., 2002, Mengistu et al., 2015) where they have the capacity to mediate Fc-effector functions in vitro (Guan et al., 2013; reviewed in Lewis et al., 2014, Pollara et al., 2013, Veillette et al., 2016). In the context of release targets these epitopes are expressed on infected/budding cells upon triggering of Env trimer with the cell surface CD4. Recent studies confirm that monoclonal Abs (mAbs) recognizing A32-like release targets exhibit considerable ADCC potency and are thus capable of eliminating infected cells, including cells budding HIV-1 (Ferrari et al., 2011, Veillette et al., 2014b; reviewed in Kramski et al., 2015, Lewis et al., 2014, Pollara et al., 2013, Veillette et al., 2016). The picture is even stronger for vaccine-induced immunity in humans, as the A32-like epitopes have been linked to protective ADCC responses in the RV144 vaccine trial (Bonsignori et al., 2012, Haynes et al., 2012). Moreover, the A32-like Abs synergized with V2-specific Abs for infectious virus capture and ADCC of both tier-1 and -2 viruses, suggesting that crosstalk between these two specificities contributed to vaccine efficacy due to FcR-effector functions (Pollara et al., 2014).

While A32 region epitopes were shown to be highly immunogenic during HIV-1 infection as they become exposed during transitional rearrangements of the Env trimer bound to CD4 on nascently or persistently infected cells, efforts to selectively construct these transitional and conformational targets as stable protein molecules have been difficult for three reasons. First, this region is buried inside the Env trimer where it is not accessible (or poorly accessible) for Ab recognition in the ligand-free closed state (Kwon et al., 2015, Ray et al., 2014, Sanders et al., 2013). Second, although the A32 region is exposed on monomeric gp120, its exposure is stabilized only upon binding soluble CD4. gp120 is highly dynamic, undergoing large conformational changes in solution (Guttman et al., 2012), and CD4 triggering is required for stable formation of these targets in monomeric gp120 (Fouts et al., 2000). Third, the Cluster A epitope region is localized exclusively within the inner domain (ID), which is known to be conformationally more mobile (Finzi et al., 2010, Guttman et al., 2012, Pancera et al., 2010) than other portions of the HIV-1 Env (such as the outer domain [OD], which has successfully been grafted and expressed independently of the ID) (Joyce et al., 2013, Yang et al., 2004). This makes efforts toward isolating this region into a minimal structural unit highly challenging.

Here, we describe the first successful attempt at isolating the ID of HIV-1 gp120 as an independent protein molecule that efficiently encapsulates conformational A32-like epitopes within a minimal structural unit of Env without the complication of other known epitope specificities. The ID design was guided by our atomic-level description of the A32 epitope region gained from the crystal structures of several A32-like Abs in complexes with CD4-triggered gp120 (Acharya et al., 2014, Gohain et al., 2015). Through two phases of structure-based design we developed ID2, which consists of the ID of the gp120 core stabilized in the CD4-bound conformation and expressed independently of the OD. ID2 stably expresses the transitional C1-C2 epitopes involved in potent FcR-effector responses to HIV-1 as indicated by physicochemical, antigenicity, and functional testing. The crystal structure of ID2 in complex with the A32 Fab confirms its proper folding and represents the first co-crystal structure of mAb A32, the canonical Ab of the ADCC Cluster A region, complexed with its cognate Env antigen.

## Results

### Design and Purification of an Independent Inner Domain Molecule, ID1

**Figure 1:**
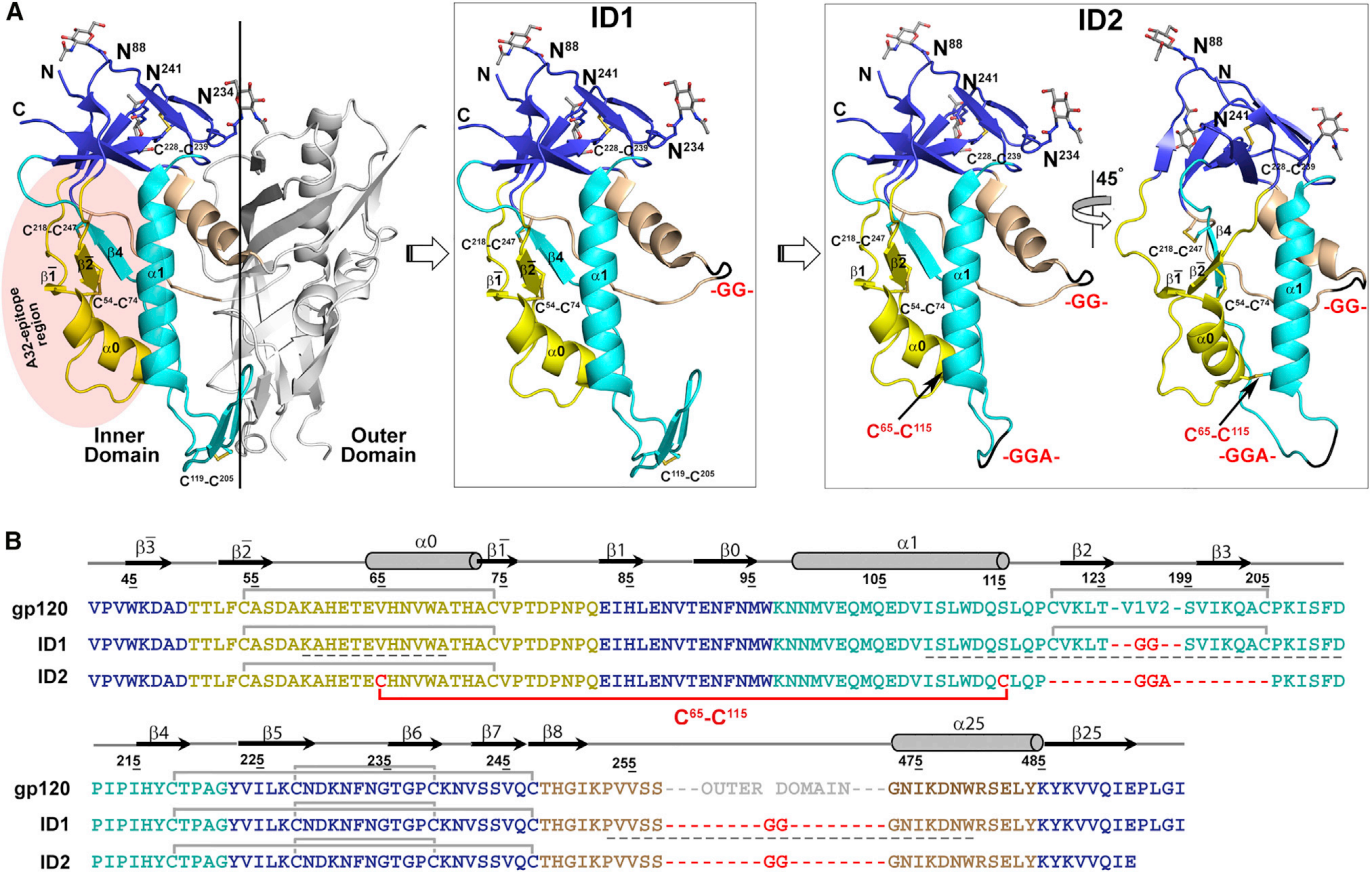
Design of Inner Domain Constructs (A) Subsequent steps of design of the independent ID molecule. Left: structure of gp120_93TH057_ core_e_ in CD4-bound conformation (from PDB code: 4H8W); middle and right: the putative ID1 and ID2 structure. “Layered” architecture of gp120 inner domain is shown with the seven-stranded β sandwich colored violet, layer 1 in yellow, layer 2 in cyan, layer 3 in light orange. The outer domain is shown in gray. Asn-proximal NAG residues of glycosyl group and disulfide bonds of ID are shown as balls and sticks. The -GG(A)- linkers are shown in black. The gp120 surface engaged in binding to the C1-C2 region-specific mAbs is shaded in red. (B) Sequence of ID constructs of 93TH057 isolate. The -GG- linkers and C_65_-C_115_ disulfide introduced to ID1 sequence are shown in red. See also Figure S1.

Our previous structural analysis of complexes formed between A32-like mAbs and the gp120 antigen indicated that A32-like mAbs invariantly recognize the epitope surface around the β$\bar{1}$, β$\bar{2}$ strands and the α0 and α1 helices of layers 1 and 2 of the ID of gp120 in its CD4-bound conformation (Acharya et al., 2014, Gohain et al., 2015). Residues of the variable loops and the OD are not involved in binding. Based on this information, we hypothesized that an independent ID molecule, with the V1V2 loop deleted and preferentially stabilized in the CD4-bound conformation, would be a suitable minimal structure for presenting A32-like epitopes with the same antigenic features as seen in gp120-CD4 complexes. Our initial design was based on an observation that the unliganded extended gp120 core (core_e_) tends to spontaneously adopt the CD4-bound conformation (Dey et al., 2009, Kwon et al., 2012). We developed a stable ID construct of clade A/E isolate 93TH057, which is composed of a gp120 core_e_ sequence with the OD sequence (residues 258–472, HxBc2 numbering) replaced within ID layer 3 by a simple -GG- dipeptide linker (Figure 1). The first design of the independent ID molecule is referred to as ID1. A synthetic ID1 gene was fused to the signal peptide sequence, placed into the pCMV6-A-puro expression vector, expressed by transient transfection of 293T cells, and purified as described in Experimental Procedures and Figure S1.

mAb A32 is capable of binding unliganded gp120, but CD4 triggering significantly enhances its exposure and stability in the context of the single-chain protein (Coutu and Finzi, 2015, Guan et al., 2013). As a prelude to testing ID1, we tested binding of Cluster A mAbs including mAb A32, and the anti-Cluster A mAbs N5-i5, N60-i3, 2.2c, and JR4 (Guan et al., 2013) to monomeric full-length BaL gp120 (gp120_BaL_) and to the single-chain gp120_BaL_-sCD4 complex (full-length single-chain [FLSC]) (Fouts et al., 2000) by surface plasmon resonance (Table 1). All mAbs tested showed increased affinities for the gp120-CD4 complex as shown by an 8- to 32-fold lower affinity constant (K_D_) for FLSC compared with unliganded gp120. In all cases, the increased affinity for the gp120_BaL_-sCD4 complex resulted from the faster association rates and reduced dissociation rates, confirming that the epitopes in the C1-C2 region are indeed inducible and stabilized in the context of gp120 triggered by CD4. Next we tested ID1 with the same Ab set to test how the C1-C2 region epitopes are preserved within the ID1 construct. As shown in Table 1, ID1 bound effectively to each of five mAbs tested, with a K_D_ value in a range of 1.1–9.3 nM, thus with affinities comparable with or slightly improved over that of gp120 (K_D_ value in a range of 2.0–12.0 nM). This indicates that ID1 preserves the antigenic properties of gp120 and is folded to display A32-like epitopes. To our knowledge, ID1 represents a first stable construct of the gp120 ID expressed independently of the OD.

**Table 1:** Binding Kinetics of mAb A32, N5-i5, N60-i3, JR4, and 2.2c to the FLSC, gp120, and ID1 as Measured by Surface Plasmon Resonance

	gp120_BaL_	FLSC	Fold Difference	ID1	Fold Difference^a^	Fold Difference^b^
**mAb A32**						

K_D_ (M) × 10^−9^	3.2 ± 0.07	0.23 ± 0.01	13.9	9.30 ± 1.10	2.9	40.4
k_a_ (1/M s) × 10^5^	1.39 ± 0.02	4.43 ± 0.01	3.2	0.85 ± 0.04	1.6	5.2
k_d_ (1/s) ×10^−5^	43.7 ± 0.99	10.2 ± 0.09	4.3	78.50 ± 5.20	1.8	7.7

**mAb N5-i5**						

K_D_ (M) × 10^−9^	5.03 ± 0.03	0.29 ± 0.14	17.3	1.10 ± 0.16	4.6	3.8
k_a_ (1/M s) × 10^5^	0.72 ± 0.01	8.25 ± 2.50	11.5	4.50 ± 1.00	6.3	1.8
k_d_ (1/s) ×10^−5^	36.0 ± 0.28	23.8 ± 13.80	1.5	48.30 ± 4.30	1.3	2.0

**mAb N60-i3**						

K_D_ (M) × 10^−9^	2.03 ± 0.07	0.25 ± 0.01	8.0	3.50 ± 0.13	1.7	13.8
k_a_ (1/M s) × 10^5^	2.53 ± 0.06	12.4 ± 0.00	4.9	4.30 ± 0.25	1.7	2.9
k_d_ (1/s) ×10^−5^	51.2 ± 0.50	31.4 ± 0.71	1.6	148 ± 2.70	2.9	4.7

**mAb JR4**						

K_D_ (M) × 10^−9^	2.34 ± 0.16	0.18 ± 0.01	13.0	6.00 ± 0.33	2.6	33.3
k_a_ (1/M s) × 10^5^	2.62 ± 0.09	6.43 ± 0.06	2.5	1.60 ± 0.10	1.6	4
k_d_ (1/s) ×10^−5^	61.1 ± 1.80	11.6 ± 0.28	5.3	94.40 ± 0.60	1.5	8.1

**mAb 2.2c**						

K_D_ (M) × 10^−9^	12.00 ± 0.07	0.50 ± 0.06	24.0	3.00 ± 0.30	4	6.0
k_a_ (1/M s) × 10^5^	0.73 ± 0.01	11.5 ± 1.40	15.7	2.70 ± 0.16	3.7	4.3
k_d_ (1/s) ×10^−5^	87.5 ± 1.10	55.6 ± 14.40	1.6	80.30 ± 2.40	1.1	1.4

a Fold difference relative to gp120.

b Fold difference relative to FLSC.

### Structural Characterization of ID1 in Complex with Anti-Cluster A mAb JR4

To assess how ID1 is folded, we determined the crystal structure of ID1 in complex with the Fab of JR4, a C1-C2-specific mAb recognizing the A32-C11 mixed epitope within the Cluster A region (Gohain et al., 2015). The structure of Fab JR4-ID1 complex was solved at 1.85 Å with one copy of complex present in the asymmetric unit of the crystal. The calculated electron density maps showed clearly defined density for the whole JR4 Fab and for 104 of 169 residues of ID1 (Table 2).

**Table 2:** Data Collection and Refinement Statistics

	Fab JR4-ID1	Fab A32-ID2_293 HEK Cells_	Fab A32-ID2_*E. coli*_
**Data Collection**			

Wavelength (Å)	0.9795	1.128	0.9795
Space group	C222(1)	P2(1)2(1)2	P2(1)2(1)2
Cell parameters			
*a*, *b*, *c* (Å)	78.5, 89.9, 180.8	75.7, 208.2, 73.2	75.8, 211.8, 72.9
α, β, γ (°)	90, 90, 90	90, 90, 90	90, 90, 90
Complexes (a.u.)	1	2	2
Resolution (Å)	50–1.85 (1.88–1.85)	50–3.10 (3.15–3.10)	50.0–3.00 (3.05–3.00)
No. of reflections			
Total	299,100	165,660	119,799
Unique	51,569	19,959	20,655
*R*_merge_^a^ (%)	10.7 (100)	18.6 (95.5)	16.3 (88.0)
*I*/σ	15.0 (1.1)	14.6 (1.3)	9.5 (1.2)
Completeness (%)	94.9 (91.4)	93.2 (96.8)	87.1 (86.7)
Redundancy	5.8 (5.7)	8.3 (8.3)	5.8 (5.8)

**Refinement Statistics**			

Resolution (Å)	50–1.85	50–3.1	50–3.02
*R*^b^ (%)	18.6	23.2	22.7
*R*_free_^c^ (%)	22.3	28.9	28.8
No. of atoms			
Protein	4,034	8,470	8,600
Water	376	0	4
Ligand/ion	32	0	0
Overall *B* value (Å^2^)			
Protein	44.7	95.8	90.2
Water	42.4	–	61.7
Ligand/ion	47.5	–	–
RMSD			
Bond lengths (Å)	0.018	0.010	0.008
Bond angles (°)	1.79	1.53	1.45
Ramachandran^d^			
Favored (%)	8.9	87.5	87.0
Allowed (%)	8.9	9.4	9.4
Outliers (%)	0.5	3.1	3.6
PDB	5FCU	4YBL	4YC2

Values in parentheses are for highest-resolution shell.

a *R*_merge_ = ∑|*I* − <*I*>|/∑*I*, where *I* is the observed intensity and <*I*> is the average intensity obtained from multiple observations of symmetry-related reflections after rejections.

b *R* = ∑‖*F*_o_| − |*F*_c_‖/∑|*F*_o_|, where *F*_o_ and *F*_c_ are the observed and calculated structure factors, respectively.

c *R*_free_ as defined by Brünger (1992).

d Calculated with MolProbity.

**Figure 2:**
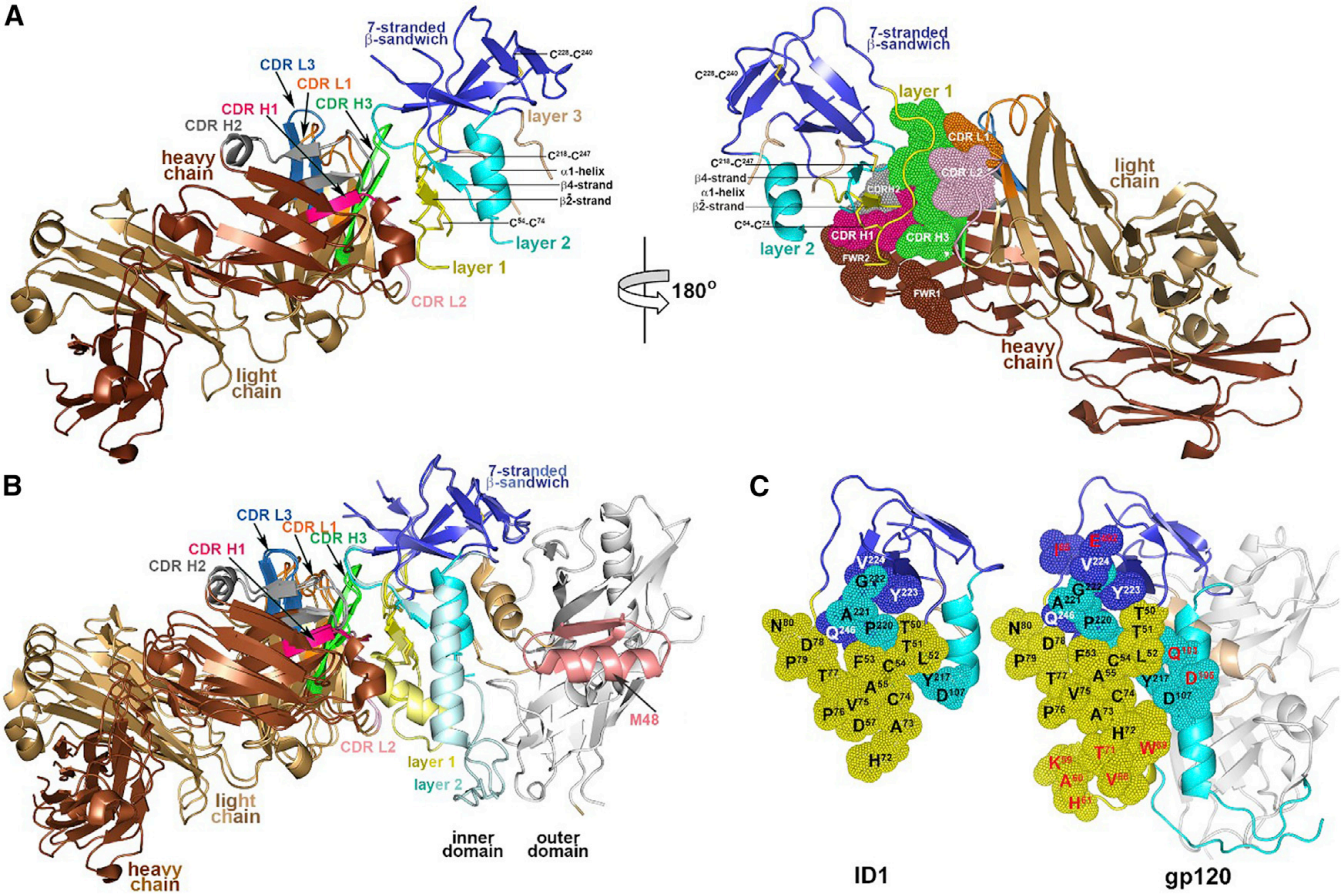
Crystal Structure of the JR4 Fab-ID1 Complex (A) The JR4 Fab-ID1 complex and its 180° view about a vertical axis are shown in a ribbon diagram with the molecular surface displayed over the residues of the Fab involved in ID1 binding (right). The structural gp120 elements engaged in JR4 Fab binding—the ${\text{β}}^{\bar{2}}$-,β4 strand and the α1 helix—are labeled. (B) Structural comparison of JR4 Fab-ID1 and JR4 Fab-gp120_93TH057_ core_e_-M48 complexes. Complexes were aligned based on the ID of gp120 and are colored in darker and lighter shades for JR4 Fab-ID1 and the JR4 Fab-gp120 interface, respectively. (C) JR4 epitope footprints on ID1 and the gp120core_e_. The gp120 residues buried at the complex interface are shown as spheres and displayed over the ribbon diagram of ID1 and the gp120core_e_. The gp120 residues that contribute to JR4 Fab binding in JR4 Fab-gp120_93TH057_ core_e_-M48 complex but are not involved in JR4 Fab binding to ID1 are shown in red. See also Figure S2 and Table S1.

The overall structure of JR4 Fab-ID1 complex is shown in Figure 2. ID1 preserves well the overall fold of the ID as in the gp120 core_e_. The seven-stranded β sandwich is fully folded, the β$\bar{2}$ and β4 strands, and three (C^54^-C^74^, C^218^-C^247^, C^228^-C^249^) out of four disulfide bonds are defined and formed as in the gp120 core_e_. On the other hand, large portions (38.5%) of the ID1 molecule are disordered in the complex and not resolved in the structure. These include sequences forming the α0 helix in the gp120 core_e_ (residues A^58^KAHETEVHNVWA^70^), the first and second -GG- linker (I^109^SLWDQSLQPCVKLT-GG-SVIKQACPKISFD^211^ and P^253^VVSS-GG-GNIKDNW^479^, including the fourth C^119^-C^205^ disulfide of the ID, respectively) and the C-terminal residues (E^492^PLGI^496^) of ID1 (Figures 1B and 2A). The JR4 Fab anchors ID1 mainly through its heavy chain (85.0% of the Fab buried surface area [BSA] of the complex) and by contacting the residues of layer 1 (75.2% of the ID1 BSA, Table S1) (Figure 2A).

To determine whether the JR4 mixed A32-C11 epitope is preserved within the ID1 molecule, we compared the JR4 Fab-ID1 complex with the previously determined crystal structure of the ternary complex formed by the JR4 Fab, the gp120 core_e_ and the CD4 mimetic M48 (Gohain et al., 2015) (Figures 2B and 2C). As shown in Figure 2B, large portions of layers 1 and 2 of the ID, forming the structural framework for the JR4 epitope within the CD4 mimetic-triggered gp120 core_e_, are disordered in the JR4 Fab-ID1 complex. These include the parts of the β$\bar{2}$-α0-connecting coil (residues A^58^KAH) and α0 helix (residues E^64^VHNVWA) of layer 1 and α1 helix (residues N^98^NMVEQMQEDV), and the β2-, β3-strand and V1V2-stem region of layer 2. In addition, the β$\bar{1}$ strand (residues A^74^CV) of layer 1, which in the gp120 core_e_ is adjacent to the α0 helix, is not formed in the ID1 molecule. Overall, the interactive surface that becomes buried due to the JR4 Fab-ID1 interaction encompasses a total of 1,930 Å^2^ (953 Å^2^ contributed by ID1 and 977 Å^2^ by Fab); this is 410 Å^2^ smaller than the interactive surface buried at JR4 Fab-gp120_93TH057_ core_e_ interface (BSA of 2,340 Å^2^ with 1,195 Å^2^ buried by gp120 core_e_ and 1,145 Å^2^ by Fab). Several layer-1 contacts are missing in the JR4-ID1 complex compared with JR4-gp120 complex, specifically the JR4 heavy chain contacts to layer 1 (Figures 2C and S2). The missing contacts can be attributed to disorder in the ID1 structure. The similarity in the A32-like Ab affinities for ID1 and gp120 suggest that the disordered residues in ID1, specifically the α0 helix and parts of the α1 helix, might mirror disorder in full-length gp120 in the absence of CD4.

### Structure-Based Design of ID2 and Evaluation of Its Antigenic Properties

Structural analysis of the Fab JR4-ID1 complex revealed that the conformational CD4i epitopes of the A32-like region are not fully formed within ID1, with regions around the V1V2 stem and α0 helix flexible and not contributing to anti-Cluster A mAb binding. We hypothesized that adding a disulfide bond at the bases of α0 and α1 helices could restore and preserve CD4i epitopes in this region (Figure 1). Accordingly, we mutated V_65_ and S_115_ to cysteines to form a single disulfide bond at the α0- and α1-helix junction. Similar mutations have been shown to increase CD4i epitope exposure in the context of full-length gp120 (Kassa et al., 2013). This revised second independent ID molecule, with the V1V2 stem removed and stabilized by a C^65^-C^115^ disulfide bond, was designated ID2.

**Figure 3:**
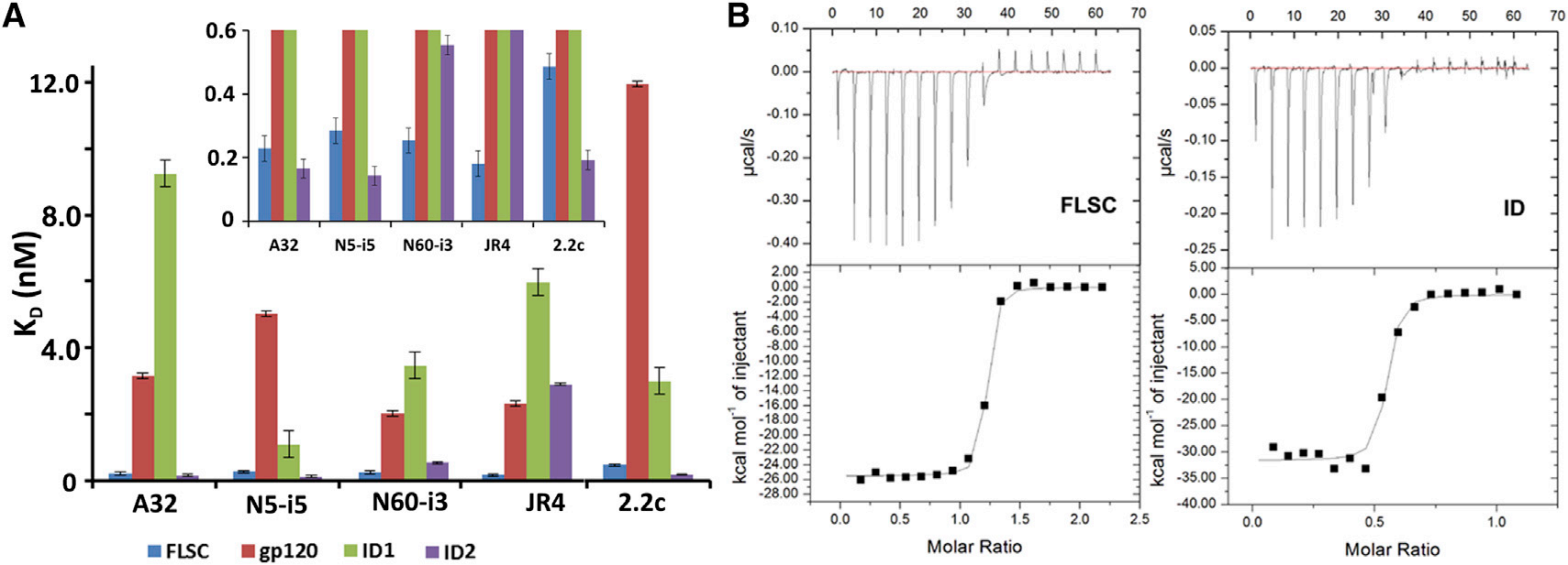
Binding of Cluster A Abs to ID2 (A) Bar graph for binding affinity (K_D_) of Cluster A mAbs to FLSC (blue), gp120_BaL_ (red), ID1 (green), and ID2 (purple). The inset represents a magnified view of the higher binding affinity section of the graph. Units are in nM. (B) ITC curves for A32 IgG against FLSC and ID2. See also Table S2.

We tested the affinity of ID2 for a panel of anti-Cluster A mAbs using surface plasmon resonance (Figure 3 and Table S2). ID2 bound the panel of mAbs tested with an average of 17-fold tighter affinities than ID1, and similar or higher than those of FLSC (Figure 3A). The increase in affinity was largely attributable to an increase in k_a_ for the complex and to a lesser extent a decrease in k_d_, suggesting that ID2 more closely resembles the CD4-bound conformation of gp120 recognized by these mAbs. To further determine that ID2 is folded to stably present the A32-like epitopes in solution, we tested the binding of mAb A32 to ID2 by isothermal titration calorimetry (ITC) and compared it with the mAb A32 interaction with FLSC (Figure 3B). These data clearly indicate that ID2 adopts a CD4-bound conformation in solution that closely resembles the presentation of the A32-epitope in CD4-trigerred gp120. The binding kinetics of mAb A32 to ID2 and FLSC are very similar, with K_D_ values of 9.0 and 11.5 nM and ΔH of −2.553 (±0.18) and −3.15 (±0.69) kcal/mol for the mAb A32-ID2 and mAb A32-FLSC interactions, respectively. Finally, we also tested whether ID2 is recognized by A32-like mAbs isolated from RV144 vaccinees (Bonsignori et al., 2012) and mouse mAbs specific for C1-linear epitopes poorly exposed within the properly folded gp120 preparations (Abacioglu et al., 1994, Moore et al., 1994b) (Table S3). Whereas RV144 mAbs bound efficiently to ID2 with affinities comparable with anti-Cluster A mAb JR4, the peptide specific mAbs B18 and C4 failed to recognize ID2. Altogether, these data confirm that ID2 stably presents the desired conformational epitopes within the C1-C2 gp120 region and shows the antigenicity profile of the CD4-triggered gp120.

### Structural Characterization of ID2 in Complex with mAb A32

**Figure 4:**
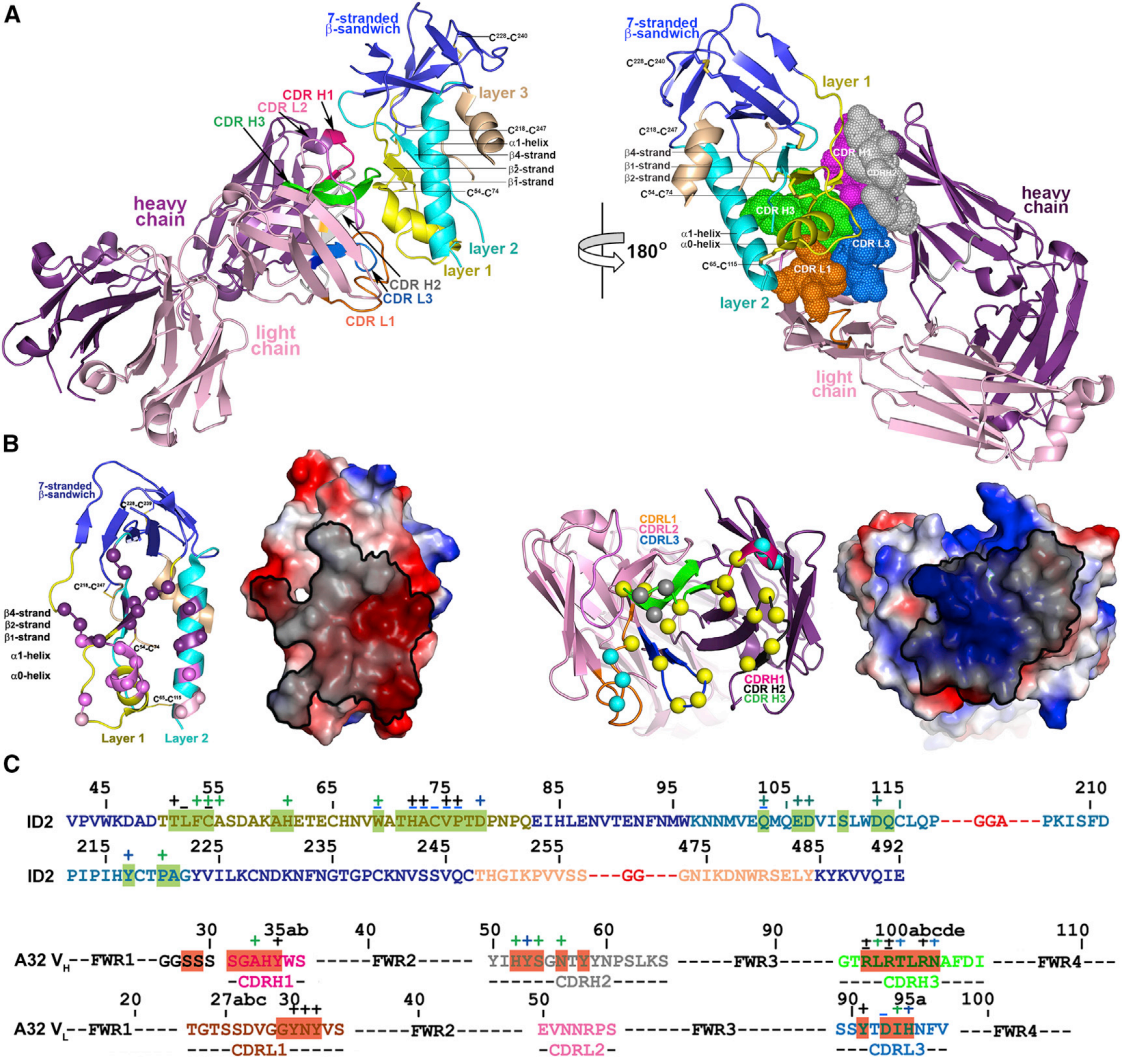
Crystal Structure of A32 Fab-ID2 Complex (A) A32-ID2 complex (left) and its 180° view about a vertical axis are shown in ribbon diagram with molecular surface displayed over the residues of the Fab involved in ID2 binding (right). See also Figure S3 and Table S1. (B) Details of the A32 Fab-ID2 interface. A32 Fab contacts on ID2 and ID2 contacts on the A32 Fab are shown as balls displayed over the ID2/A32 Fab ribbon diagram and highlighted in black over the ID2/A32 Fab electrostatic potential surface. The A32 Fab contacts are shown in dark purple, light purple, and purple for heavy, light, and both chain(s) contacts, respectively. The ID2 contacts through residues of layer 1, layer 2, and both are shown in yellow, cyan, and gray, respectively. (C) A32 Fab and ID2 contact residues on the primary sequence of ID2 and A32 Fab, respectively. Residues contributing to the A32 Fab-ID2 interface as defined by PISA (Krissinel and Henrick, 2007) are highlighted, and contacts as defined by a 5-Å cutoff are marked above the sequence. Side-chain (+) and main-chain (−) contacts are colored based on contact type; hydrophobic in green, hydrophilic in blue, or both in black.

To confirm whether ID2 preserves the Cluster A epitopes of CD4-triggered gp120 better than ID1, we determined its crystal structure in complex with mAb A32 Fab (Experimental Procedures; Table 2; Figure 4 and S3). The structural characterization of the ID2 complex fully validated its design. As shown in Figure 4A, ID2 fully preserves the fold of the ID of the gp120 core_e_ in its CD4-bound conformation with the seven-stranded β sandwich and all the secondary structural elements of layers 1 and 2 (including α0 and α1 helices) present and arranged to form the C1-C2 epitope as seen in complexes of A32-like mAbs with CD4-triggered gp120 cores_e_ (Acharya et al., 2014; Figures 1 and 4A). Most of the ID2 molecule is defined in the complex (85% of the molecule, compared with 61% for ID1, Figure S4) with the only unresolved regions mapping to the N and C termini (residues 42–44 and 492) and to the introduced flexible -GGA- and -GG- linkers (residues 117–118, 206–209, 254–256, and 472–474). Each of the four disulfide bonds (including the newly introduced C^65^-C^115^ disulfide bond) are defined and maintained as predicted. The structural alignment of ID2 from the Fab A32-ID2 complex with the gp120 core_e_ (from Fab N5-i5-gp120_93TH057_ core_e_-d1d2CD4; Acharya et al., 2014) and with ID1 resulted in a root-mean-square deviation (RMSD) between main-chain atoms of 0.76 Å (523 atoms) and 1.36 Å^2^ (416 atoms), respectively, indicating a closer similarity of the overall structure of ID2 to the ID in the CD4-triggered gp120 cores_e_ bound to A32-like mAbs, than to ID1.

The A32 Fab-ID2 complex also represents the first co-crystal structure of mAb A32 with its cognate gp120 antigen. mAb A32, similar to A32-like mAbs N5-i5, JR4, and N60-i3 described previously in complexes with gp120 cores_e_, binds exclusively within the C1-C2 regions of gp120 (Figure 4B). Its epitope is formed by bridging mobile layers 1 and 2 with anchoring points centered on the ${\text{β}}^{\bar{2}}$ strand, the α0 helix, and ${\text{β}}^{\bar{1}}$ strand, and the α1 helix (residues 51–54, 60–61, 69, and 71–78 [layer 1] and 103, 106–107, 110, 113–114, 217, and 220–221 [layer 2]) (Figures 4B and 4C). The interactive surface that becomes buried at the Fab A32-ID2 interface is 1,701 Å^2^ (856 Å^2^ contributed by Fab and 845 Å^2^ by ID2, Table S1). Most of the Fab contacts are contributed by its heavy chain, with complementarity-determining region (CDR) H3 making contacts to the ${\text{β}}^{\bar{2}}$ strand of gp120 (residues T^51^LFC) and CDR H1–3 contacting the residues of the α0 helix and the ${\text{β}}^{\bar{1}}$ strand (e.g., W^69^ATHACVP motif). In contrast, contacts to the α1 helix of layer 2 are almost exclusively contributed by two arginines of CDR H3 (R^99^ and R^100B^) with some minor contributions from the CDR L1, CDR H1, and the FWR1 (Figures 4B and 4C). The A32 paratope is electropositive largely due to three arginines, R^97^, R^99^, and R^100B^, which make up 75% of the CDR H3 BSA and approximately 50% of the heavy chain BSA (Table S1). In addition to complementing the electronegative gp120 epitope, these three electropositive residues simultaneously bind layers 1 and 2 and stabilize the gp120 conformation in much the same way as the C^65^-C^115^ disulfide bond.

**Figure 5:**
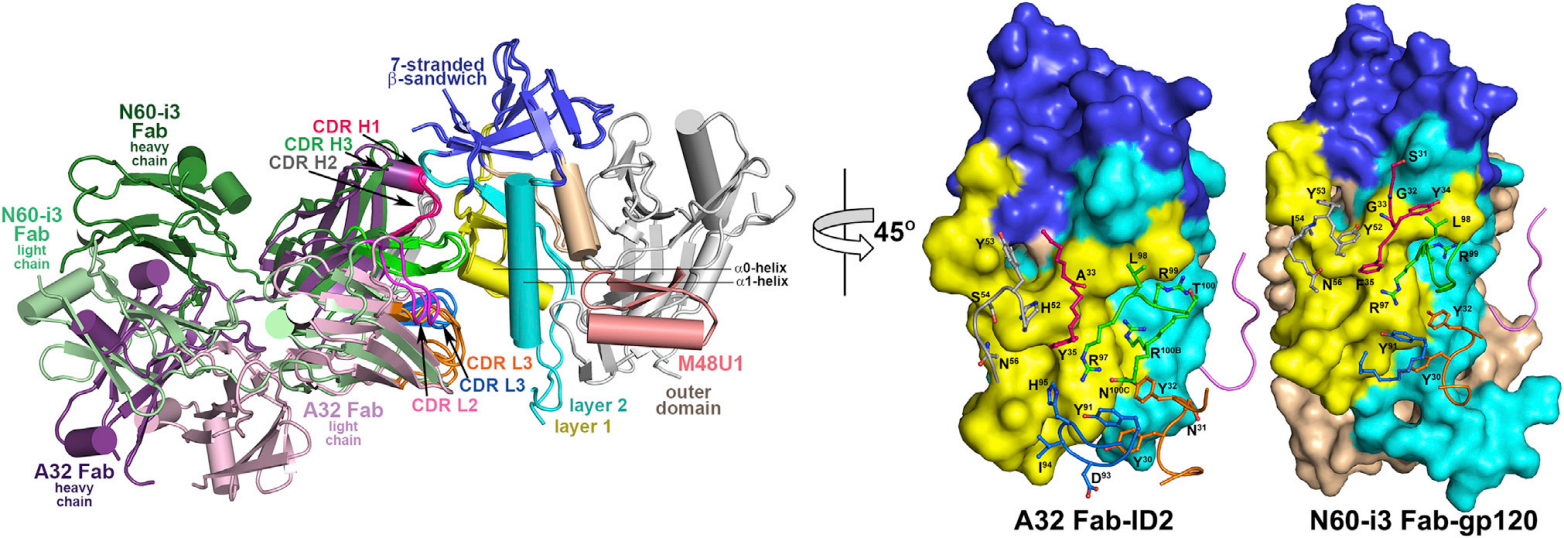
Comparison of A32 Fab-ID2 and N60-i3 Fab-gp120_93TH057_core_e_-M48U1 Complexes Complexes superimposed based on the gp120 ID are shown with light/heavy chain of A32 Fab and N60-i3 Fab is colored in light/dark purple and green, respectively. A 45° view shows the details of A32 Fab-ID2 and N60-i3-gp120core_e_. The molecular surfaces are displayed over the ID2 and gp120core_e_ surfaces, and CDRs of Fabs with residues contributing to the binding are shown.

Of the four anti-Cluster A Abs characterized to date in complexes with gp120 (Acharya et al., 2014, Gohain et al., 2015), mAb A32 shares the most similar mode of antigen recognition with mAb N60-i3. mAb A32 and N60-i3 share 78% and 83% sequence identity in their heavy- and light-chain variable (V) domains, respectively (70.1% identity for the CDRs alone) and recognize gp120 with striking similarities (Figure 5). They approach the antigen through the same angle and utilize the same CDR contacts to interact with their cognate epitopes. The close structural similarity of the Fab-antigen interfaces is reflected in a relatively low RMSD value of 1.80 Å^2^ calculated for main-chain atoms of the V domains of A32 and N60-i3 and ID2 and the corresponding gp120 core_e_ residues, respectively. The major difference between mAb A32 and N60-i3 and other A32-like mAbs is the stronger reliance of mAb A32 on its interaction with the α1 helix in layer 2 of the ID (Figure 5 and Table S1).

### ID2 Fully Preserves Functional Epitopes of the C1-C2 Region of gp120

**Figure 6:**
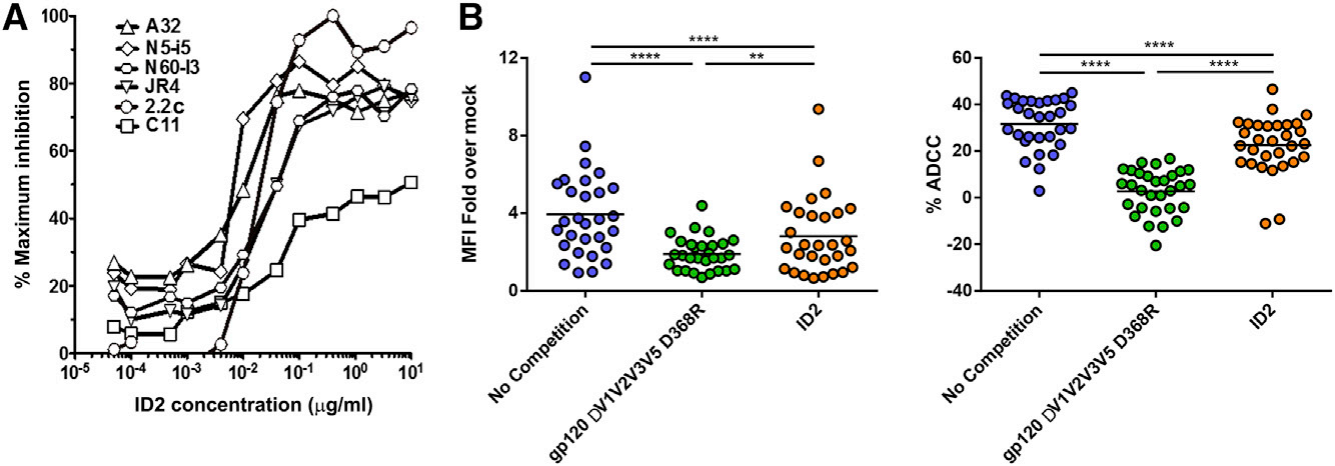
Functional Properties of ID2 (A) ID2-competition of Fc-mediated effector functions of anti-Cluster A mAbs. The activities of Cluster A mAbs (at concentrations corresponding to IC_75_) in (RF)-ADCC assay were measured in the presence of increasing concentrations of ID2 protein using gp120-coated EGFP-CEM-NKr-CCR5-SNAP cells as targets and PBMCs as effector cells. (B) ADCC competition of Abs in sera from HIV-infected individuals. CEM.NKr cells infected with NL4.3 ADA.GFP Nef^−^Vpu^−^ virus were used at 48 hr post-infection for surface staining of Abs bound (left) and an RF^−^ADCC assay (right) using sera from HIV-1-infected individuals pre-incubated in absence or presence of 10 μg/μl ΔV1V2V3V5 D368R or 2.66 μg/μl ID2 for 30 min at room temperature. Data shown are representative of at least three different experiments. Statistical significance was tested using paired one-way ANOVA (^∗∗^p < 0.01, ^∗∗∗∗^p < 0.0001).

ID2 represents the first stable design of the gp120 ID expressed independently of the OD and stabilized in a CD4-bound confirmation to exclusively express the non-neutralizing, ADCC epitopes of the C1-C2 region. The binding analysis indicates that anti-Cluster A mAbs bind to ID2 with affinities comparable with that of the gp120-CD4 complex; thus the desired epitopes are preserved within the ID2 design (Figure 3). Additionally, to test whether ID2 engrafts epitopes involved in Fc-effector functions directed to the C1-C2 region of HIV-1 Env, we performed a competition assay in which effector functions of anti-Cluster A mAbs were tested at their effective concentrations in the presence of increasing concentrations of ID2. The assay was performed using two complementary methods: the rapid fluorimetric (RF)-ADCC assay (Gomez-Roman et al., 2006), which measures trogocytosis (e.g., Fc-effector function mediated by CD14^+^ monocytes) rather than ADCC (Kramski et al., 2012), and the flow cytometry (fluorescence-activated cell sorting [FACS])-based ADCC assay, which allows the quantification of cell killing by ADCC (e.g., mediated by natural killer [NK] cells) (Richard et al., 2014). As shown in Figure 6A, ID2 completely inhibited of all of the A32-like mAbs tested in the (RF)-ADCC assay and as well as the mixed A32-C11 mAb JR4 at a concentration range of 0.1–10 μg/ml. The Fc-effector activity of mAb C11, which recognizes an epitope involving residues of N and C termini and seven-stranded β sandwich of gp120 (Gohain et al., 2015) not present within the ID design, was minimally affected. The same pattern of inhibition of mAb A32 and C11 was observed in the FACS-based ADCC assay (Figure S4). Together, these data confirm that ID2 is folded to stably display functional A32-like and mixed A32-C11-like epitopes within the ADCC Cluster A region.

Next, we tested how efficiently ID2 can adsorb Abs present in HIV^+^ sera and compete with their ADCC activities. It was shown previously that infected individuals frequently elicit Abs specific for A32-like epitopes and that the predominant fraction of the ADCC response in sera from HIV-1-infected individuals is directed at epitopes exposed on the CD4-triggered gp120 core including the A32 region (e.g., according to Ferrari et al., [2011], pre-incubation of target cells with Fabs of A32 and 17b blocked about 50% of the ADCC activity in 7 of 14 HIV-1-infected individuals). As shown in Figure 6B, we pre-incubated HIV^+^ sera (20 samples of sera derived from HIV-1-infected individuals including both progressors and long-term non-progressors as characterized in Table S4) with either a D368R gp120 dV1V2V3V5 mutant or ID2 protein before being assayed for their ability to bind to CEM.NKr cells infected with a Nef^−^Vpu^−^ virus (Figure 6B, left) and to mediate ADCC (Figure 6B, right). We used viruses lacking Nef and Vpu (i.e., presenting high levels of CD4 and Env at the cell surface) to ensure effective exposure of A32-like targets on the infected cell surface as described by Ferrari et al. (2011) and Veillette et al. (2015). The D368R gp120 dV1V2V3V5 mutant, bearing CD4i epitopes of gp120 core, was previously shown to almost completely abrogate cell-surface staining and ADCC of Nef-Vpu-infected cells (Ding et al., 2015, Veillette et al., 2015). ID2 specifically bound Abs present in HIV^+^ sera and adsorbed approximately 30% of total ADCC activity. The levels of observed ADCC competition are with agreement with previous observations indicating that A32 epitopes constitute a large fraction but not the only fraction of ADCC targets in sera of infected individuals (Ferrari et al., 2011, Veillette et al., 2015). Altogether these data indicate that ID2 stably engrafts the epitopes recognized by A32-like Abs involved in ADCC from the sera of HIV-1-infected individuals.

## Discussion

The A32-like epitopes of the Cluster A region map exclusively within the inner domain of the gp120 (Acharya et al., 2014, Gohain et al., 2015), on the gp120 face that interacts with the gp41 ectodomain in the trimeric structure of the HIV-1 Env (Pancera et al., 2014, Yang et al., 2003, York and Nunberg, 2004). On the native trimer of most HIV-1 isolates, this region is buried at the trimer interface and is inaccessible for Ab recognition until the interaction with the target cell receptor and, possibly, the co-receptor (reviewed in Lewis et al., 2015, Veillette et al., 2016). Our recent studies indicate that substantial structural rearrangements of the ID are required to convert to the conformation recognized by A32-like Abs from the Ab-inaccessible native, untriggered trimeric Env (Acharya et al., 2014). Moreover, accumulating evidence suggests that exposure of these epitopes on the virion spike strictly depends on binding cell surface anchored CD4, since triggering with soluble CD4 does not lead to exposure of these epitope targets on virion-associated HIV-1 trimers (Acharya et al., 2014, Mengistu et al., 2015, Ray et al., 2014).

In this report we describe a new molecule, ID2, which consists of the ID of HIV-1 gp120 expressed independently of the OD and stabilized to effectively express the conformational C1-C2 region epitopes. To our knowledge, we are the first to successfully isolate the ID of gp120 as an independent and fully stable molecule. In the past, the isolation of this gp120 region into a minimal structural unit was thought to be highly challenging or impossible, mostly due to the fact that the ID is highly mobile and undergoes pronounced conformational rearrangements during the structural transitions of the HIV-1 Env trimer in the viral entry process (Pancera et al., 2014, Yang et al., 2003). A stable ID construct of HIV-1 gp120 was developed, expressed in both mammalian and bacterial systems, and characterized by structural and functional means. Through two phases of rational structure-based design, we obtained the ID2 molecule, which displays the A32-like epitopes within a minimal structural unit of gp120 consisting of 167 residues and constrained by the engineered C_65_-C_115_ bond to adopt a CD4-bound state. ID2 stably expresses the C1-C2 epitopes as indicated by significantly increased or comparable affinities for anti-Cluster A mAbs compared with unliganded gp120 or gp120-CD4 complexes, respectively. Structural analysis of ID complexes with A32 and JR4 mAbs fully validated the ID design. ID1, the first ID design, consisting of ID as in gp120 core_e_s (Dey et al., 2009, Kwon et al., 2012), failed to adopt the CD4-bound conformation required for formation of CD4i epitopes of A32-like region. ID1 displayed antigenic properties of full-length untriggered gp120 and the epitope of anti-Cluster A mAb JR4 was only partially preserved within its design, as indicated by large portions of the JR4 binding surface being disordered in the Fab JR4-ID1 complex. Regions of the ID1 molecule found to be disordered in the Fab JR4-ID1 complex were previously shown to undergo a significant conformational change upon CD4 binding, evident in the comparisons of CD4-bound gp120 core_e_ structures (Acharya et al., 2014, Kwon et al., 2012) with the recent gp140 SOSIP trimers (Julien et al., 2013, Lyumkis et al., 2013, Pancera et al., 2014). We were able to eliminate the ID1 disorder and fully induce the CD4i A32-like region in the ID2 design that incorporates truncations of the V1V2 stem region and a new C_65_-C_115_ disulfide bond. The C_65_-C_115_ bond links the α0 (layer 1) and α1 (layer 2) helices and thus stabilizes the ID in the CD4-bound conformation. We show here the ID2 construct, which is based on the clade A/E 93TH057 virus sequence, but we were also able to obtain stable ID2 preparations using sequences of other clades including clade B strain YU2. Both of these preparations preserve antigenic properties of ID2 of clade A/E 93TH057 (Table S5), confirming that the ID2 design is a durable structural template and can be used to generate other independent ID molecules based on sequences of any HIV-1 strain.

ID2 abrogates entirely Fc-mediated effector function of A32 and A32-like mAbs and specifically adsorbs ADCC activities against target CEM.NKr T cells infected with Nef^−^Vpu^−^ NL4.3 virus from the sera of HIV-1-infected individuals. It was shown previously that CD4i Env epitopes, including targets within the A32 region, are preferentially recognized by Abs capable of potent ADCC present in sera (Ferrari et al., 2011, Richard et al., 2014, Veillette et al., 2014a, Veillette et al., 2014b, Veillette et al., 2015) or cervico-vaginal lavages (Batraville et al., 2014) of HIV-1-infected individuals. ID2 is therefore folded to stably display the functional epitopes of the C1-C2 region. It is designed to engraft non-neutralizing A32-like epitopes selectively without any other known epitopes, especially those involved in neutralizing Ab response. By design ID2 consists of two faces: a face harboring the C1-C2 epitopes and a face exposed by the removal of the OD that does not harbor any known epitope targets (Figure 1). As a minimal structural unit of gp120 effectively expressing the A32-like region, ID2 has significant translational value because it can be employed as a specific probe for the analysis of Ab responses to these non-neutralizing epitope targets (e.g., in immune correlates analysis of future vaccine trials or epitope mapping). It also constitutes a novel immunogen candidate, selectively and stably presenting the ADCC A32-like epitopes that could be used in challenge studies to address the exclusive role of non-neutralizing Abs in vaccine protection. We previously showed that many of the residues forming the Cluster A epitope are highly conserved among HIV isolates (Acharya et al., 2014, Gohain et al., 2015, Guan et al., 2013), indicating that ADCC responses specific for this region will be cross-reactive and undergo limited immune escape. As such, they represent an important vaccine target. Anti-Cluster A mAbs also share similar characteristics in their V domains, with moderate length CDR H3 loops and a low degree of V_H_ chain maturation (Acharya et al., 2014, Gohain et al., 2015, Guan et al., 2013). Thus, ID2 can potentially be used to elicit anti-Cluster A Abs in vivo without intensive immunization regimens and without immunogens designed and optimized to recognize an affinity-matured Ab variant. Studies are under way to evaluate ID2 as an immunogen capable of selective induction of the A32-like responses in animal hosts.

Finally, our studies are the first to define the structure of the epitope of mAb A32, the canonical mAb of the Cluster A region. The previous reports were based solely on binding studies with Env mutants associated the A32 epitope primarily with the C1 region of gp120 (Finzi et al., 2010, Moore et al., 1994a, Pancera et al., 2010). Our Fab A32-ID2 complex represents the first co-crystal structure of mAb A32 with its gp120 antigen and permits the precise description of the gp120-binding footprint of mAb A32. Accordingly, mAb A32 recognizes a conformational epitope formed by the ${\text{β}}^{\bar{2}}$ strand, the α0 helix and ${\text{β}}^{\bar{1}}$ strand, and the α1 helix of the gp120 ID. mAb A32 binds to gp120, almost entirely overlapping the footprint and mimicking the binding mode of the mAb N60-i3, a human Ab isolated from an HIV-1-infected individual of our natural virus suppressor cohort (Gohain et al., 2015). Thus, it recognizes the same discontinuous site within the C1-C2 region as other anti-Cluster A mAbs described previously to be capable of potent FcR-effector function against gp120-coated and virus-bound target cells (Acharya et al., 2014). The A32 epitope, like epitopes of other anti-Cluster A mAbs (Acharya et al., 2014, Gohain et al., 2015), is buried inside the HIV-1 Env trimer where it is not accessible (or poorly accessible) for Ab recognition in the ligand-free closed state (Mengistu et al., 2015, Ray et al., 2014, Sanders et al., 2002, Sanders et al., 2013). Thus, our work provides a structural understanding of why mAb A32 does not efficiently recognize and inactivate (through an ADCC mechanism) target cells infected with wild-type HIV-1 viruses. The HIV-1 accessory viral protein U (Vpu) and negative regulatory factor (Nef) protein, two well-established regulators of cell-surface CD4 expression, reduce the levels of CD4 on the surface of the infected target (reviewed in Veillette et al., 2016). In the absence of CD4, the HIV-1 Env trimers remain in the ligand-free closed state, inhibiting the effective recognition of infected cells by mAb A32 (Richard et al., 2015, Veillette et al., 2014b, Veillette et al., 2015). Utilization of the full potential of mAb A32 and other Abs targeting A32 region epitopes to inactivate infected cells through an FcR mechanism in vivo will be possible only if effective strategies capable of diminishing the CD4-downregulation effect are developed and implemented.

## Experimental Procedures

### Inner Domain Design

ID1 consists of residues 44–123 (HxBc2 numbering) and 199–256 of gp120 connected by a glycine-glycine linker that is, in turn, connected to the C terminus of gp120 (residues 472–496) by another glycine-glycine linker to remove the OD. ID2 was made based on the ID1 sequence in which the V1V2 region (residues 118–206) was replaced by a two-glycine alanine linker. ID2 was further stabilized by adding an extra disulfide by mutating V^65^ and S^115^ to cysteines. Initial clones were synthesized with optimized codons for mammalian expression using the native gp120 leader sequence. Mutations were added with the Quikchange PCR mutagenesis kit (Stratagene) as per the manufacturer's protocol.

### Protein Expression and Purification

IDs were expressed by transfection using the FreeStyle 293T and GnT1^−^ 293T Mammalian Expression System (Invitrogen) for functional and structural analysis, respectively, and according to the protocol provided by the manufacturer. IDs were purified using an N5-i5 IgG affinity column. N5-i5 IgG was chemically crosslinked to protein A resin using the Pierce Protein A IgG Plus orientation kit (Thermo Fisher Scientific). Protein bound to the N5-i5 IgG affinity column was eluted with 0.1 M glycine (pH 3.0) and immediately diluted 10:1 with 1 M Tris-HCl (pH 8.5).

ID2 for co-crystallization studies was grown in the Origami (DE) *Escherichia coli* strain (Novagen). ID2 sequence was cloned into the pMal-c5e expression vector (New England Biolabs) with an N-terminal maltose-binding protein (MBP)-thioredoxin tag followed by a six-histidine tag and a thrombin cleavage sequence. The cell lysate (after 2–3 min of sonication and centrifugation at 12,000 × *g* for 30 min) was loaded onto a HiTrap nickel column (GE Healthcare). MBP-thioredoxin-ID2 was eluted with 25 mM Tris-HCl (pH 8.0) and 500 mM imidazole (pH 8.0). The MBP-thioredoxin tag was removed by digestion overnight at 4°C with agarose-linked bovine thrombin (Sigma) and passing the lysate over an amylose column. Flow-through fractions were concentrated and purified further using an N5-i5 IgG affinity column as described above.

### Crystallization and Data Collection

For structural studies the ID proteins were deglycosylated with 10 units/μg of Endo Hf (NE Biolabs) for 3–4 hr at 37°C. Endo Hf was removed by passing the sample over the column of amylose resin (NE Biolabs).

Fab fragments were generated from IgG by papain cleavage using papain-linked agarose slurry (G Biosciences). Fabs were purified by passing the digest over a HiTrap protein A column (GE Healthcare), followed by gel filtration chromatography in a Hiload Superdex200 16/60 column (GE Healthcare) equilibrated with 5 mM Tris-HCl (pH 8.5) and 150 mM NaCl.

Fab-ID complexes for crystallization experiments were made by mixing purified Fab and ID protein at a 1:1.5 M ratio and incubating on ice for 30 min. Complexes were purified by gel filtration chromatography in a Hiload Superdex200 16/60 column (GE Healthcare) equilibrated with 5 mM Tris-HCl (pH 8.5) and 150 mM NaCl. Crystals were grown by the hanging-drop method. Combinations of Fabs of Cluster A mAbs A32, N5-i5, N60-I3, and JR4 were used to prepare complexes with ID1 and ID2 and tested for crystal formation. Only complexes forming diffraction-quality crystals, which include Fab JR4-ID1 and Fab A32-ID2 complex, were selected for further analysis. Fab JR4-ID1 complex crystals were grown from 20% polyethylene glycol (PEG) momomethyl ether, 0.2 M ammonium sulfate, and 0.1 M Tris-HCl (pH 7.5). Fab A32-ID2 complex crystals were grown from 18% to 22% PEG 6000 or PEG 8000 and 0.1 M Tris-HCl (pH 8.5). JR4 and A32 complex crystals were flash-frozen in liquid nitrogen after a brief soak in the crystallization condition supplemented with 20% and 15% MPD, respectively.

Diffraction data were collected at the Stanford Synchrotron Radiation Light Source at the beam lines BL12-2 (Fab JR4-ID1 and Fab A32-ID2_E. coli_) and BL7-1 (Fab A32-ID2_293 cells_), equipped with MAR325, PILATUS 6M PAD, and ADSC Quantum 315 area detectors, respectively. All data were processed and reduced with HKL 2000 (Otwinowski et al., 1997).

Structures were solved by molecular replacement with Phaser (McCoy, 2007) from the CCP4 suite based on the coordinates of gp120 (PDB: 4H8W), and the JR4 Fab (PDB: 4RFE) and A32 Fab (PDB: 3TNM). Refinement was carried out with Refmac (Murshudov et al., 1997) and Phenix (Adams et al., 2002). Refinement was coupled with manual refitting and rebuilding with Coot (Emsley and Cowtan, 2004). The first Fab A32-ID2 structure was solved using ID2 grown in 293HEK cells. The structure was refined to an R_c_/R_free_ of 23.2/28.9 with several residues of the complex missing and not built into a model due to the lack of interpretable experimental densities (Table 2). New crystals were grown with ID2 expressed from an *E. coli* expression system to improve the quality of the diffraction data. These crystals diffracted to a resolution of 3.0 Å and had the same crystallographic space group as crystal form 1, but were not isomorphous. Well-defined experimental densities obtained from these data allowed us to unambiguously build 133 out of 156 residues of ID2 in the complex (Table 2 and Figure S4).

### Surface Plasmon Resonance Analysis

The binding affinity and kinetics of ID constructs to mAb A32, N5-i5, N60-i3, JR4, and 2.2c were assessed by surface plasmon resonance on a Biacore T-100 (GE Healthcare) at 25°C. The kinetic constants were determined using a 1:1 Langmuir model of binding with BIAevaluation software (GE Healthcare).

### ITC Analysis

ITC was used to characterize the interaction of FLSC and ID2 with A32 IgG using an iTC200 instrument (GE Healthcare). Titrations were performed at 25°C. Heats of dilutions were measured and subtracted from each dataset. All data were analyzed using Origin 7.0 software.

### Competition of Fc-mediated Effector Function of Anti-Cluster A Abs with ID2

Measurements were performed using the RF-ADCC assay (Gomez-Roman et al., 2006) and FACS-based assay (Richard et al., 2014). In the RF-ADCC assay, EGFP-CEM-NKr-CCR5-SNAP target cells were stained with SNAP-Surface Alexa Fluor 647 (NE Biolabs) with or without HIV-1_Bal_ gp120 (50 μg/ml). mAbs tested (at fixed concentrations corresponding to their IC_75_ value) were mixed with ID2 (serially diluted 1:3, from 10 μg/ml to 0.5 ng/ml) and incubated with gp120-sensitized targets for 15 min at room temperature. The human effector peripheral blood mononuclear cells (PBMCs, obtained from healthy donors) were added at a ratio of 50:1 (effector/target cell). After 2 hr of incubation at 37°C in 5% CO_2_, cells were washed, fixed in 1% paraformaldehyde, and samples were collected on a BD Fortessa Special Order instrument (BD Biosciences) and analyzed using FlowJo software (Tree Star).

### Serum Adsorption and ADCC Competition Assays

Written informed consent was obtained from all study participants (the Montreal Primary HIV Infection Cohort), and research adhered to the ethical guidelines of CRCHUM and was reviewed and approved by the CRCHUM institutional review board (ethics committee). Cells and viruses (NL4.3 ADA.GFP Nef^−^Vpu^−^) were used as previously described to infect CEM.NKr cells (Richard et al., 2014, Veillette et al., 2014b, Veillette et al., 2015). Sera derived from HIV-1-infected individuals, both progressers and long-term non-progressors as described and characterized in Table S4, were pre-incubated for 30 min at room temperature with either purified soluble D368R dV1V2V3V5 gp120 or ID protein at a concentration of 10 μg or 2.66 μg protein/μl of serum, respectively, before being assayed for their ability to bind to infected cells and mediate their ADCC-dependent elimination as previously described (Richard et al., 2014, Veillette et al., 2014b, Veillette et al., 2015).

## Author Contributions

W.D.T., N.G., G.L., and M.P. designed, performed research, and analyzed the data; M.V., J.-P.C., G.L., and A.F. performed serum adsorption and ADCC competition assays; C.O., M.L.V., M.E., A.L.D., and T.R.F. performed experiments related to ID functional characterization; W.D.T., N.G., and M.P. wrote the paper with all authors providing comments or revisions.

## Accession Numbers

The accession numbers for the crystal structures reported in this paper are: PDB: 5FCU for Fab JR4-ID1 complex, PDB: 4YBL for Fab A32-ID2_293 HEK cells_ complex, and PDB: 4YC2 for Fab A32-ID2_*E. coli*_.

## Supplemental Information

Document S1. Figures S1–S4 and Tables S1–S5

Document S2. Article plus Supplemental Information

## References

[bib1] Abacioglu Y.H., Fouts T.R., Laman J.D., Claassen E., Pincus S.H., Moore J.P., Roby C.A., Kamin-Lewis R., Lewis G.K. (1994). Epitope mapping and topology of baculovirus-expressed HIV-1 gp160 determined with a panel of murine monoclonal antibodies. AIDS Res. Hum. Retroviruses.

[bib2] Acharya P., Tolbert W.D., Gohain N., Wu X., Yu L., Liu T., Huang W., Huang C.C., Kwon Y.D., Louder R.K. (2014). Structural definition of an antibody-dependent cellular cytotoxicity response implicated in reduced risk for HIV-1 infection. J. Virol..

[bib3] Adams P.D., Grosse-Kunstleve R.W., Hung L.W., Ioerger T.R., McCoy A.J., Moriarty N.W., Read R.J., Sacchettini J.C., Sauter N.K., Terwilliger T.C. (2002). PHENIX: building new software for automated crystallographic structure determination. Acta Crystallogr. D Biol. Crystallogr..

[bib4] Batraville L.A., Richard J., Veillette M., Labbe A.C., Alary M., Guedou F., Kaufmann D.E., Poudrier J., Finzi A., Roger M. (2014). Short communication: anti-HIV-1 envelope immunoglobulin Gs in blood and cervicovaginal samples of Beninese commercial sex workers. AIDS Res. Hum. Retroviruses.

[bib5] Bonsignori M., Pollara J., Moody M.A., Alpert M.D., Chen X., Hwang K.K., Gilbert P.B., Huang Y., Gurley T.C., Kozink D.M. (2012). Antibody-dependent cellular cytotoxicity-mediating antibodies from an HIV-1 vaccine efficacy trial target multiple epitopes and preferentially use the VH1 gene family. J. Virol..

[bib6] Brünger A.T. (1992). Free R value: a novel statistical quantity for assessing the accuracy of crystal structures. Nature.

[bib7] Chung A.W., Ghebremichael M., Robinson H., Brown E., Choi I., Lane S., Dugast A.S., Schoen M.K., Rolland M., Suscovich T.J. (2014). Polyfunctional Fc-effector profiles mediated by IgG subclass selection distinguish RV144 and VAX003 vaccines. Sci. Transl. Med..

[bib8] Coutu M., Finzi A. (2015). HIV-1 gp120 dimers decrease the overall affinity of gp120 preparations for CD4-induced ligands. J. Virol. Methods.

[bib9] Dey B., Svehla K., Xu L., Wycuff D., Zhou T., Voss G., Phogat A., Chakrabarti B.K., Li Y., Shaw G. (2009). Structure-based stabilization of HIV-1 gp120 enhances humoral immune responses to the induced co-receptor binding site. PLoS Pathog..

[bib10] Ding S., Veillette M., Coutu M., Prevost J., Scharf L., Bjorkman P.J., Ferrari G., Robinson J.E., Sturzel C., Hahn B.H. (2015). A highly-conserved residue of the HIV-1-gp120 inner domain is important for ADCC responses mediated by anti-cluster a antibodies. J. Virol..

[bib12] Emsley P., Cowtan K. (2004). Coot: model-building tools for molecular graphics. Acta Crystallogr. D Biol. Crystallogr..

[bib13] Ferrari G., Pollara J., Kozink D., Harms T., Drinker M., Freel S., Moody M.A., Alam S.M., Tomaras G.D., Ochsenbauer C. (2011). An HIV-1 gp120 envelope human monoclonal antibody that recognizes a C1 conformational epitope mediates potent antibody-dependent cellular cytotoxicity (ADCC) activity and defines a common ADCC epitope in human HIV-1 serum. J. Virol..

[bib14] Finnegan C.M., Berg W., Lewis G.K., DeVico A.L. (2001). Antigenic properties of the human immunodeficiency virus envelope during cell-cell fusion. J. Virol..

[bib15] Finnegan C.M., Berg W., Lewis G.K., DeVico A.L. (2002). Antigenic properties of the human immunodeficiency virus transmembrane glycoprotein during cell-cell fusion. J. Virol..

[bib16] Finzi A., Xiang S.H., Pacheco B., Wang L., Haight J., Kassa A., Danek B., Pancera M., Kwong P.D., Sodroski J. (2010). Topological layers in the HIV-1 gp120 inner domain regulate gp41 interaction and CD4-triggered conformational transitions. Mol. Cell.

[bib17] Fouts T.R., Tuskan R., Godfrey K., Reitz M., Hone D., Lewis G.K., DeVico A.L. (2000). Expression and characterization of a single-chain polypeptide analogue of the human immunodeficiency virus type 1 gp120-CD4 receptor complex. J. Virol..

[bib18] Fouts T.R., Bagley K., Prado I.J., Bobb K.L., Schwartz J.A., Xu R., Zagursky R.J., Egan M.A., Eldridge J.H., LaBranche C.C. (2015). Balance of cellular and humoral immunity determines the level of protection by HIV vaccines in rhesus macaque models of HIV infection. Proc. Natl. Acad. Sci. U S A.

[bib19] Gohain N., Tolbert W.D., Acharya P., Yu L., Liu T., Zhao P., Orlandi C., Visciano M.L., Kamin-Lewis R., Sajadi M.M. (2015). Cocrystal structures of antibody N60-i3 and antibody JR4 in complex with gp120 define more cluster a epitopes involved in effective antibody-dependent effector function against HIV-1. J. Virol..

[bib20] Gomez-Roman V.R., Florese R.H., Patterson L.J., Peng B., Venzon D., Aldrich K., Robert-Guroff M. (2006). A simplified method for the rapid fluorometric assessment of antibody-dependent cell-mediated cytotoxicity. J. Immunol. Methods.

[bib21] Guan Y., Pazgier M., Sajadi M.M., Kamin-Lewis R., Al-Darmarki S., Flinko R., Lovo E., Wu X., Robinson J.E., Seaman M.S. (2013). Diverse specificity and effector function among human antibodies to HIV-1 envelope glycoprotein epitopes exposed by CD4 binding. Proc. Natl. Acad. Sci. U S A.

[bib22] Guttman M., Kahn M., Garcia N.K., Hu S.L., Lee K.K. (2012). Solution structure, conformational dynamics, and CD4-induced activation in full-length, glycosylated, monomeric HIV gp120. J. Virol..

[bib23] Haynes B.F., Gilbert P.B., McElrath M.J., Zolla-Pazner S., Tomaras G.D., Alam S.M., Evans D.T., Montefiori D.C., Karnasuta C., Sutthent R. (2012). Immune-correlates analysis of an HIV-1 vaccine efficacy trial. N. Engl. J. Med..

[bib24] Jia M., Li D., He X., Zhao Y., Peng H., Ma P., Hong K., Liang H., Shao Y. (2013). Impaired natural killer cell-induced antibody-dependent cell-mediated cytotoxicity is associated with human immunodeficiency virus-1 disease progression. Clin. Exp. Immunol..

[bib25] Joyce M.G., Kanekiyo M., Xu L., Biertumpfel C., Boyington J.C., Moquin S., Shi W., Wu X., Yang Y., Yang Z.Y. (2013). Outer domain of HIV-1 gp120: antigenic optimization, structural malleability, and crystal structure with antibody VRC-PG04. J. Virol..

[bib26] Julien J.P., Cupo A., Sok D., Stanfield R.L., Lyumkis D., Deller M.C., Klasse P.J., Burton D.R., Sanders R.W., Moore J.P. (2013). Crystal structure of a soluble cleaved HIV-1 envelope trimer. Science.

[bib27] Kassa A., Dey A.K., Sarkar P., Labranche C., Go E.P., Clark D.F., Sun Y., Nandi A., Hartog K., Desaire H. (2013). Stabilizing exposure of conserved epitopes by structure guided insertion of disulfide bond in HIV-1 envelope glycoprotein. PLoS One.

[bib28] Kramski M., Schorcht A., Johnston A.P., Lichtfuss G.F., Jegaskanda S., De Rose R., Stratov I., Kelleher A.D., French M.A., Center R.J. (2012). Role of monocytes in mediating HIV-specific antibody-dependent cellular cytotoxicity. J. Immunol. Methods.

[bib29] Kramski M., Stratov I., Kent S.J. (2015). The role of HIV-specific antibody-dependent cellular cytotoxicity in HIV prevention and the influence of the HIV-1 Vpu protein. AIDS.

[bib30] Krissinel E., Henrick K. (2007). Inference of macromolecular assemblies from crystalline state. J. Mol. Biol..

[bib31] Kwon Y.D., Finzi A., Wu X., Dogo-Isonagie C., Lee L.K., Moore L.R., Schmidt S.D., Stuckey J., Yang Y., Zhou T. (2012). Unliganded HIV-1 gp120 core structures assume the CD4-bound conformation with regulation by quaternary interactions and variable loops. Proc. Natl. Acad. Sci. U S A.

[bib11] Kwon Y., Pancera M., Acharya P., Georgiev I.S., Crooks E.T., Gorman J., Joyce M.G., Guttman M., Ma X., Narpala S. (2015). Crystal structure, conformational fixation and entry-related interactions of mature ligand-free HIV-1 Env. Nat. Struct. Mol. Biol..

[bib32] Lewis G.K., Guan Y., Kamin-Lewis R., Sajadi M., Pazgier M., Devico A.L. (2014). Epitope target structures of Fc-mediated effector function during HIV-1 acquisition. Curr. Opin. HIV AIDS.

[bib33] Lewis G.K., Finzi A., DeVico A.L., Pazgier M. (2015). Conformational masking and receptor-dependent unmasking of highly conserved Env epitopes recognized by non-neutralizing antibodies that mediate potent ADCC against HIV-1. Viruses.

[bib34] Lyumkis D., Julien J.P., de Val N., Cupo A., Potter C.S., Klasse P.J., Burton D.R., Sanders R.W., Moore J.P., Carragher B. (2013). Cryo-EM structure of a fully glycosylated soluble cleaved HIV-1 envelope trimer. Science.

[bib35] McCoy A.J. (2007). Solving structures of protein complexes by molecular replacement with Phaser. Acta Crystallogr. D Biol. Crystallogr..

[bib36] Mengistu M., Ray K., Lewis G.K., DeVico A.L. (2015). Antigenic properties of the human immunodeficiency virus envelope glycoprotein gp120 on virions bound to target cells. PLoS Pathog..

[bib37] Moore J.P., McCutchan F.E., Poon S.W., Mascola J., Liu J., Cao Y., Ho D.D. (1994). Exploration of antigenic variation in gp120 from clades A through F of human immunodeficiency virus type 1 by using monoclonal antibodies. J. Virol..

[bib38] Moore J.P., Sattentau Q.J., Wyatt R., Sodroski J. (1994). Probing the structure of the human immunodeficiency virus surface glycoprotein gp120 with a panel of monoclonal antibodies. J. Virol..

[bib39] Murshudov G.N., Vagin A.A., Dodson E.J. (1997). Refinement of macromolecular structures by the maximum-likelihood method. Acta Crystallogr. D Biol. Crystallogr..

[bib40] Otwinowski Z., Minor W., Carter C.W. (1997). Processing of X-ray diffraction data collected in oscillation mode. Methods Enzymol.

[bib41] Pancera M., Majeed S., Ban Y.E., Chen L., Huang C.C., Kong L., Kwon Y.D., Stuckey J., Zhou T., Robinson J.E. (2010). Structure of HIV-1 gp120 with gp41-interactive region reveals layered envelope architecture and basis of conformational mobility. Proc. Natl. Acad. Sci. U S A.

[bib42] Pancera M., Zhou T., Druz A., Georgiev I.S., Soto C., Gorman J., Huang J., Acharya P., Chuang G.Y., Ofek G. (2014). Structure and immune recognition of trimeric pre-fusion HIV-1 Env. Nature.

[bib43] Pollara J., Bonsignori M., Moody M.A., Pazgier M., Haynes B.F., Ferrari G. (2013). Epitope specificity of human immunodeficiency virus-1 antibody dependent cellular cytotoxicity [ADCC] responses. Curr. HIV Res..

[bib44] Pollara J., Bonsignori M., Moody M.A., Liu P., Alam S.M., Hwang K.K., Gurley T.C., Kozink D.M., Armand L.C., Marshall D.J. (2014). HIV-1 vaccine-induced C1 and V2 Env-specific antibodies synergize for increased antiviral activities. J. Virol..

[bib45] Ray K., Mengistu M., Yu L., Lewis G.K., Lakowicz J.R., DeVico A.L. (2014). Antigenic properties of the HIV envelope on virions in solution. J. Virol..

[bib46] Rerks-Ngarm S., Pitisuttithum P., Nitayaphan S., Kaewkungwal J., Chiu J., Paris R., Premsri N., Namwat C., de Souza M., Adams E. (2009). Vaccination with ALVAC and AIDSVAX to prevent HIV-1 infection in Thailand. N. Engl. J. Med..

[bib47] Richard J., Veillette M., Batraville L.A., Coutu M., Chapleau J.P., Bonsignori M., Bernard N., Tremblay C., Roger M., Kaufmann D.E. (2014). Flow cytometry-based assay to study HIV-1 gp120 specific antibody-dependent cellular cytotoxicity responses. J. Virol. Methods.

[bib48] Richard J., Veillette M., Brassard N., Iyer S.S., Roger M., Martin L., Pazgier M., Schon A., Freire E., Routy J.P. (2015). CD4 mimetics sensitize HIV-1-infected cells to ADCC. Proc. Natl. Acad. Sci. U S A.

[bib49] Sanders R.W., Vesanen M., Schuelke N., Master A., Schiffner L., Kalyanaraman R., Paluch M., Berkhout B., Maddon P.J., Olson W.C. (2002). Stabilization of the soluble, cleaved, trimeric form of the envelope glycoprotein complex of human immunodeficiency virus type 1. J. Virol..

[bib50] Sanders R.W., Derking R., Cupo A., Julien J.P., Yasmeen A., de Val N., Kim H.J., Blattner C., de la Pena A.T., Korzun J. (2013). A next-generation cleaved, soluble HIV-1 Env trimer, BG505 SOSIP.664 gp140, expresses multiple epitopes for broadly neutralizing but not non-neutralizing antibodies. PLoS Pathog..

[bib51] Tomaras G.D., Ferrari G., Shen X., Alam S.M., Liao H.X., Pollara J., Bonsignori M., Moody M.A., Fong Y., Chen X. (2013). Vaccine-induced plasma IgA specific for the C1 region of the HIV-1 envelope blocks binding and effector function of IgG. Proc. Natl. Acad. Sci. U S A.

[bib52] Veillette M., Coutu M., Richard J., Batraville L.A., Desormeaux A., Roger M., Finzi A. (2014). Conformational evaluation of HIV-1 trimeric envelope glycoproteins using a cell-based ELISA assay. J. Vis. Exp..

[bib53] Veillette M., Desormeaux A., Medjahed H., Gharsallah N.E., Coutu M., Baalwa J., Guan Y., Lewis G., Ferrari G., Hahn B.H. (2014). Interaction with cellular CD4 exposes HIV-1 envelope epitopes targeted by antibody-dependent cell-mediated cytotoxicity. J. Virol..

[bib54] Veillette M., Coutu M., Richard J., Batraville L.A., Dagher O., Bernard N., Tremblay C., Kaufmann D.E., Roger M., Finzi A. (2015). The HIV-1 gp120 CD4-bound conformation is preferentially targeted by antibody-dependent cellular cytotoxicity-mediating antibodies in sera from HIV-1-infected individuals. J. Virol..

[bib55] Veillette M., Richard J., Pazgier M., Lewis G.K., Parsons M.S., Finzi A. (2016). Role of HIV-1 envelope glycoproteins conformation and accessory proteins on ADCC responses. Curr. HIV Res..

[bib56] Wren L.H., Chung A.W., Isitman G., Kelleher A.D., Parsons M.S., Amin J., Cooper D.A., Stratov I., Navis M., Kent S.J. (2013). Specific antibody-dependent cellular cytotoxicity responses associated with slow progression of HIV infection. Immunology.

[bib57] Yang X., Mahony E., Holm G.H., Kassa A., Sodroski J. (2003). Role of the gp120 inner domain beta-sandwich in the interaction between the human immunodeficiency virus envelope glycoprotein subunits. Virology.

[bib58] Yang X., Tomov V., Kurteva S., Wang L., Ren X., Gorny M.K., Zolla-Pazner S., Sodroski J. (2004). Characterization of the outer domain of the gp120 glycoprotein from human immunodeficiency virus type 1. J. Virol..

[bib59] Yates N.L., Liao H.X., Fong Y., Decamp A., Vandergrift N.A., Williams W.T., Alam S.M., Ferrari G., Yang Z.Y., Seaton K.E. (2014). Vaccine-induced Env V1-V2 IgG3 correlates with lower HIV-1 infection risk and declines soon after Vaccination. Sci. Transl. Med..

[bib60] York J., Nunberg J.H. (2004). Role of hydrophobic residues in the central ectodomain of gp41 in maintaining the association between human immunodeficiency virus type 1 envelope glycoprotein subunits gp120 and gp41. J. Virol..

