# Supplementary material for: Paring Down HIV Env: Design and Crystal Structure of a Stabilized Inner Domain of HIV-1 gp120 Displaying a Major ADCC Target of the A32 Region
Source: Structure. 2016 May 3;24(5):697–709. doi: 10.1016/j.str.2016.03.005 (PMC4856543; doi:10.1016/j.str.2016.03.005)
Supplement: Document S1. Figures S1–S4 and Tables S1–S5 [file mmc1.pdf]

Structure, Volume *24*

Supplemental Information

Paring Down HIV Env: Design and Crystal Structure of a Stabilized Inner Domain of HIV-1 gp120 Displaying a Major ADCC Target of the A32 Region

William D. Tolbert, Neelakshi Gohain, Maxime Veillette, Jean-Philippe Chapleau, Chiara Orlandi, Maria L. Visciano, Maryam Ebadi, Anthony L. DeVico, Timothy R. Fouts, Andrés Finzi, George K. Lewis, and Marzena Pazgier

Table S1, related to Figure 2 and 4. Details of the JR4-ID1 and A32-ID2 interfaces as calculated by the EBI PISA server (http://www.ebi.ac.uk/msd-srv/prot_int/cgi-bin/piserver).

		Fab JR4-ID1	Fab A32-ID2<br>293 HEK cells	Fab A32-ID2<br>*E. coli*
**Buried Surface Area, Å²**	**gp120 total**	**953.3**	**845.1 (849.2)**	**843.8(856.5)**
	7-stranded β-sandwich	84.7	0	0
	Layer 1	716.6	633.4 (603.6)	660.8(630.2)
	Layer 2	148.4	211.6 (245.6)	183.0 (226.3)
	Layer 3	3.6	0	0
	**Heavy chain total**	**830.3**	**618.6 (609.8)**	**624.1(617.1)**
	FWR	129.3	16.1 (21.5)	11.0(23.7)
	CDR H1	209.6	107.4 (112.4)	98.0 (109.0)
	CDR H2	111.1	88.0 (73.7)	95.4 (82.1)
	CDR H3	380.3	407.1 (402.1)	419.7(402.4)
	**Light chain total**	**146.4**	**252.1 (244.8)**	**231.9 (235.5)**
	FWR	41.6	0	0
	CDR L1	49.9	133.9 (140.3)	123.7 (128.1)
	CDR L2	54.9	0	0
	CDR L3	0	118.2 (104.5)	108.2 (107.4)
	**Heavy and light chain total**	**976.8**	**870.7 (854.6)**	**855.9 (852.6)**

Table S2, related to Figure 3. Binding kinetics of mAb A32, N5-i5, N60-i3, JR4 and 2.2c to the FLSC, gp120 and ID2 as measured by SPR. The assay was run by passing the FLSC (Fouts et al., 2000), monomeric full length $gp120_{BaL}$ or ID2 over the immobilized antibody at 0-200 nM concentrations as described in Experimental Procedures. The binding kinetics (association rates (k_a), dissociation rates (k_d), and affinity constants (K_D)) were calculated with the BIAevaluation software. Standard deviations of k_a, k_d and K_D for two experiments are shown.

93TH057	$gp120_{BaL}$	FLSC	fold difference	ID2 C_{65}-C_{115}	fold difference[1]	fold diference[2]
**mAb A32**						
K_D (M) x 10^{-9}	3.2 ± 0.07	0.23 ± 0.01	**13.9**	0.17 ± 0.01	**18.8**	**1.4**
k_a(1/Ms) x 10^5	1.39 ± 0.02	4.43 ± 0.01	**3.2**	16.5 ± 0.6	**11.9**	**3.7**
k_d(1/s) x10^{-5}	43.7 ± 0.99	10.2 ± 0.09	**4.3**	27.5 ± 2.0	**1.6**	**2.7**
**mAb N5-i5**						
K_d (M) x 10^{-9}	5.03 ± 0.03	0.29 ± 0.14	**17.3**	0.14 ± 0.01	**35.9**	**2.1**
k_a(1/Ms) x 10^5	0.72 ± 0.01	8.25 ± 2.50	**11.5**	8.1 ± 0.2	**11.3**	**1.0**
k_d(1/s) x10^{-5}	36.0 ± 0.28	23.8 ± 13.80	**1.5**	11.6 ± 0.4	**3.1**	**2.1**
**mAb N60-i3**						
K_D (M) x 10^{-9}	2.03 ± 0.07	0.25 ± 0.01	**8.0**	0.55 ± 0.01	**3.7**	**2.2**
k_a(1/Ms) x 10^5	2.53 ± 0.06	12.4 ± 0.00	**4.9**	8.9 ± 0.1	**3.5**	**1.4**
k_d(1/s) x10^{-5}	51.2 ± 0.50	31.4 ± 0.71	**1.6**	49.1 ± 0.8	**1.0**	**1.6**
**mAb JR4**						
K_D (M) x 10^{-9}	2.34 ± 0.16	0.18 ± 0.01	**13.0**	2.9 ± 0.1	**1.2**	**16.1**
k_a(1/Ms) x 10^5	2.62 ± 0.09	6.43 ± 0.06	**2.5**	6.5 ± 0.6	**2.5**	**1.0**
k_d(1/s) x10^{-5}	61.1 ± 1.80	11.6 ± 0.28	**5.3**	190.0 ± 9.9	**3.1**	**16.4**
**mAb 2.2c**						
K_D (M) x 10^{-9}	12.00 ± 0.07	0.50 ± 0.06	**24.0**	0.19 ± 0.02	**63.1**	**2.6**
k_a(1/Ms) x 10^5	0.73 ± 0.01	11.5 ± 1.40	**15.7**	8.8 ± 0.28	**12.1**	**1.3**
k_d(1/s) x10^{-5}	87.5 ± 1.10	55.6 ± 14.40	**1.6**	17.0 ± 2.6	**5.1**	**3.3**

[1] fold change relative to gp120, [2] fold change relative to FLSC

Table S3, related to Figure 3. Binding kinetics of mAb C4 and B18, both recognizing linear epitopes in C1 region (Abacioglu et al., 1994) and antibodies CH38 and CH91, both recognizing conformational epitopes in C1 region and isolated from RV144 vaccinees (Bonsignori et al., 2012). The assay was run by passing the ID2 over the immobilized antibody at 0-200 nM concentrations as described in the Experimental Procedures. The binding kinetics (association rates (k_a), dissociation rates (k_d), and affinity constants (K_D)) were calculated with the BIAevaluation software. Standard deviations of k_a, k_d and K_D for two experiments are shown.

	**ID2**
**mAb CH38**	
K_D (M) x 10^{-9}	8.5 ± 2.4
k_a(1/Ms) x 10^5	1.8 ± 0.4
k_d(1/s) x10^{-5}	150 ± 11
**mAb CH91**	
K_D (M) x 10^{-9}	2.9 ± 0.16
k_a(1/Ms) x 10^5	3.1 ± 0.19
k_d(1/s) x10^{-5}	89 ±0.4
**mAb B18**	
K_D (M) x 10^{-9}	N.D.
k_a(1/Ms) x 10^5	
k_d(1/s) x10^{-5}	
**mAb C4**	
K_D (M) x 10^{-9}	N.D.
k_a(1/Ms) x 10^5	
k_d(1/s) x10^{-5}	

Table S4, related to Figure 6B. Characteristics of HIV-infected sera donors

Donor ID	Characteristics: CD4 Count (cells / mm^3)	Characteristics: Viral Load (copies / ml)
Progressors (0-24 months PI)		
1	431	43938
2	730	67190
3	600	249442
4	790	22045
5	460	180763
6	260	150963
7	785	39032
8	670	58475
9	263	60
10	592	13520
11	421	617860
12	n.d.	25626
13	1029	37793
14	647	961
	LTNPs	
15	692	50
16	514	744
17	n.d.	n.d.
18	650	50
19	680	1760
20	670	40
21	740	14547
22	590	7503
23	597	5454
24	1290	50
25	740	767
26	920	702
27	800	7206
28	810	42
29	780	949
30	480	793

PI : post infection
n.d. : no data available
LTNP: long-term non-progressor

Table S5, related to Figure 3. Binding kinetics of mAb A32, N5-i5, N60-i3, JR4 and 2.2c to the FLSC, $ID2_{93TH057}$ and $ID2_{YU2}$ as measured by SPR. The assay was run by passing the FLSC (Fouts et al., 2000), $ID2_{93TH057}$ or $ID2_{YU2}$ over the immobilized antibody at 0-200 nM concentrations as described in the Experimental Procedures. The binding kinetics (association rates (k_a), dissociation rates (k_d), and affinity constants (K_D)) were calculated with the BIAevaluation software. Standard deviations of k_a, k_d and K_D for two experiments are shown.

	**FLSC**	$ID2_{93TH057}$	**fold difference**	**$ID2_{YU2}$**	**fold difference [1]**	**fold difference[2]**
**mAb A32**						
K_D (M) x 10^{-9}	0.23 ± 0.01	0.17 ± 0.01	**1.4**	0.8 ± 0.02	**3.5**	**4.7**
k_a(1/Ms) x 10^5	4.43 ± 0.01	16.5 ± 0.6	**3.7**	3.5 ± 0.09	**1.3**	**4.7**
k_d(1/s) x10^{-5}	10.2 ± 0.09	27.5 ± 2.0	**2.7**	27.9 ± 1.5	**2.7**	**1.0**
**mAb N5-i5**						
K_D (M) x 10^{-9}	0.29 ± 0.14	0.14 ± 0.01	**2.1**	0.23 ± 0.02	**1.3**	**1.6**
k_a(1/Ms) x 10^5	8.25 ± 2.50	8.1 ± 0.2	**1.0**	3.5 ± 0.04	**2.4**	**2.3**
k_d(1/s) x10^{-5}	23.8 ± 13.80	11.6 ± 0.4	**2.1**	7.7 ± 0.9	**3.1**	**1.5**
**mAb N60-i3**						
K_D (M) x 10^{-9}	0.25 ± 0.01	0.55 ± 0.01	**2.2**	3.5 ± 0.03	**14**	**6.4**
k_a(1/Ms) x 10^5	12.4 ± 0.00	8.9 ± 0.1	**1.4**	2.9 ± 0.06	**4.3**	**3.1**
k_d(1/s) x10^{-5}	31.4 ± 0.71	49.1 ± 0.8	**1.6**	101.5 ± 3.2	**3.2**	**2.1**
**mAb JR4**						
K_D (M) x 10^{-9}	0.18 ± 0.01	2.9 ± 0.1	**16.1**	4.8 ± 0.2	**26.7**	**1.7**
k_a(1/Ms) x 10^5	6.43 ± 0.06	6.5 ± 0.6	**1.0**	2.2 ± 0.01	**2.9**	**3.0**
k_d(1/s) x10^{-5}	11.6 ± 0.28	190.0 ± 9.9	**16.4**	103.6 ± 5.5	**8.9**	**1.8**
**mAb 2.2c**						
K_D (M) x 10^{-9}	0.50 ± 0.06	0.19 ± 0.02	**2.6**	79.6 ± 2.1	**159**	**419**
k_a(1/Ms) x 10^5	11.5 ± 1.40	8.8 ± 0.28	**1.3**	0.27 ± 0.01	**42.6**	**32.5**
k_d(1/s) x10^{-5}	55.6 ± 14.40	17.0 ± 2.6	**3.3**	217 ± 3.4	**3.9**	**12.8**

[1] fold change relative to FLSC, [2] fold change relative to $ID2_{93TH057}$

Initially we incorporated the sequence of clade A/E isolate 93TH057 into the ID2 design. To test if the C1-C2 epitopes would be preserved within the ID scaffold if sequences of other HIV-1 clades were used we designed and expressed the clade B YU2 equivalent of ID2. mAb A32, N5-i5, N60-i3 and JR4 show affinities for $ID2_{YU2}$ comparable to $ID2_{93TH057}$ (fold change in KD value between 1.7 to 6.4) while binding of mAb 2.2c is markedly affected by a His 61 to Tyr sequence change in YU2. We have shown previously that mAb 2.2c recognizes exclusively residues of layer 1. Taken together, these binding studies indicate that the ID2 construct adopts the CD4-bound conformation in solution with substantially increased affinities for Cluster A mAbs.

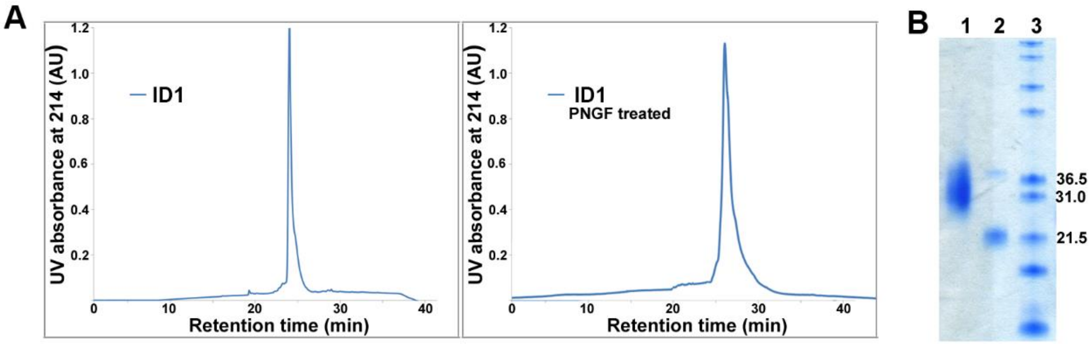

Figure S1, related to Figure 1. Physico-chemical characterization of ID1 (**A**) Analytical RP-HPLC. ID1 (left panel) and PNGF digestion product of ID1 (right panel) were analyzed on a Symmetry 300^{TM} C_4 column (2.1 x 150 mm, 3.5μm) using a linear gradient of 5-65% of acetonitrile at a flow rate of 0.25 mL/min over 30 min. (**B**) SDS PAGE analysis of the purified ID1 (line 1), ID1 subjected to cleavage with PNG F (line 2), protein standards (line 3).

The typical production and purification procedure yielded 1-2 mg of ID1 per liter of culture medium. ID1 was analyzed by RP-HPLC, SDS-PAGE and gel filtration chromatography. The ID1 construct contains eight cysteine residues and all eight are involved in disulfide bond formation. A single RP-HPLC protein peak (Panel A) indicates that the ID1 molecule, folded, fully oxidized and purified, consists of a homogeneous species in solution and not a mixture of different disulfide-bonded isomers. Purified ID1 protein has a molecular weight of ~31-32 kDa as determined by SDS-PAGE (Panel B). This corresponds to the molecular weight of the protein backbone (MW 21,886 Da, as calculated on the basis of the average isotopic compositions of the protein) and 3 N-glycosyl groups attached to N88, N234 and N241. Removal of these glycosyl groups with PNGF yielded a protein backbone of molecular weight in good agreement with the expected values calculated on the basis of the average isotopic compositions of the protein (line 2). Analysis of the ID sequence identified no new potential sites of N- or O-linked glycosylation in the newly exposed solvent accessible surface after the OD removal.

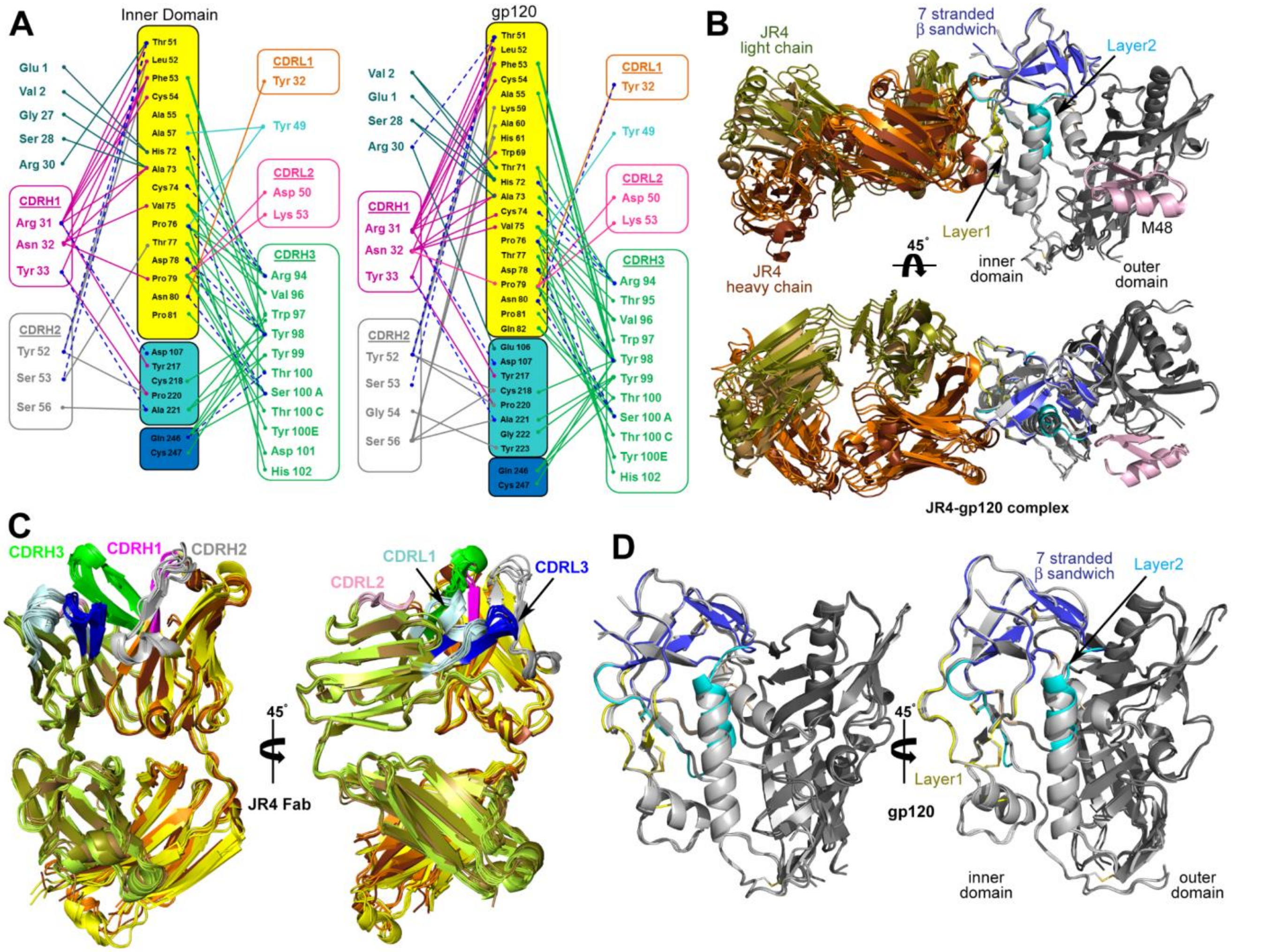

Figure S2, related to Figure 2. Structural comparison of JR4 Fab-ID1 and JR4 Fab-gp120$_{93TH057}$ core$_e$-M48 complexes (**A**) Network of interactions formed between the JR4 Fab and the ID1 (left) and JR4 Fab and gp120 core$_e$ (right) and as defined by a 5 Å distance criteria cutoff. H-bonds are shown as dashes in blue. The complementarity-determining regions (CDRs) are shown in orange (CDR L1), pink (CDR L2), blue (CDR L3), magenta (CDR H1), grey (CDR H2), and green (CDR H3). The gp120 ID is shown with the 7-stranded β-sandwich colored violet, layer 1 in yellow and layer 2 in cyan. A total of 55 (including 11 H-bonds) contacts as defined by a 5 Å cutoff are formed at the JR4 heavy/light chain–ID1 interface as compared to 54 (15 H-bonds) contacts at the JR4 heavy/light chain– gp120$_{93TH057}$ core$_e$ interface (**B**) Ribbon diagram of a superposition of the JR4 Fab-ID1 complex and two copies of JR4 Fab-gp120$_{93TH057}$ core$_e$-M48 complex present in the asymmetric unit of the crystal (Gohain et al., 2015). The complexes were superimposed based on the gp120 ID. The light/heavy chain of JR4 Fab is shown in chocolate/sand and orange/olive for the JR4 Fab-ID1 and JR4 Fab-gp120$_{93TH057}$ core$_e$-M48 complex, respectively. The gp120 core$_e$ OD is colored in dark grey and the ID is shown in light grey. ID1 is colored in a "layered" color scheme with the 7-stranded β-sandwich blue, layer 1 yellow and layer 2 cyan. The mimetic peptides M48 is colored in pink. The structural alignment of the JR4 Fab-ID1 complex to each of two copies of JR4 Fab-gp120$_{93TH057}$ core$_e$-M48 complex resulted in the average root mean square deviation (RMSD) of 1.41 Å for 2112 main chain atoms of the complex (1696 atoms of JR4 Fab and 416 atoms of inner domain). The root mean square deviation (RMSD) between complexes for main chain atoms is 1.3 Å. **(C)** Ribbon diagram of a superposition of the JR4 Fab from the JR4 Fab-ID1 complex (chocolate/sand), two copies of the JR4 Fab from the JR4 Fab-gp120$_{93TH057}$ core$_e$-M48 complex (orange/olive) and the apo JR4 Fabs (yellow/limon). The average RMSD for JR4 Fab molecules was 1.51 and 1.59 Å for JR4 Fab-ID1/JR4 Fab-gp120$_{93TH057}$ core$_e$-M48 and JR4 Fab-ID1/apo JR4 Fab alignments, respectively. (**D**) Ribbon diagram of a superposition (based on the ID) of the ID1 from JR4 Fab-ID1 complex (colored in as in **B**) and two copies of the gp120 core$_e$ from JR4 Fab-gp120$_{93TH057}$ core$_e$-M48 complex (light/dark grey for inner and outer domain, respectively). The disulfide bonds in the ID are shown as yellow sticks and labelled. The RMSD for 416 atoms of the ID to the gp120$_{93TH057}$ core$_e$s ranged from 1.37 to 1.4 Å.

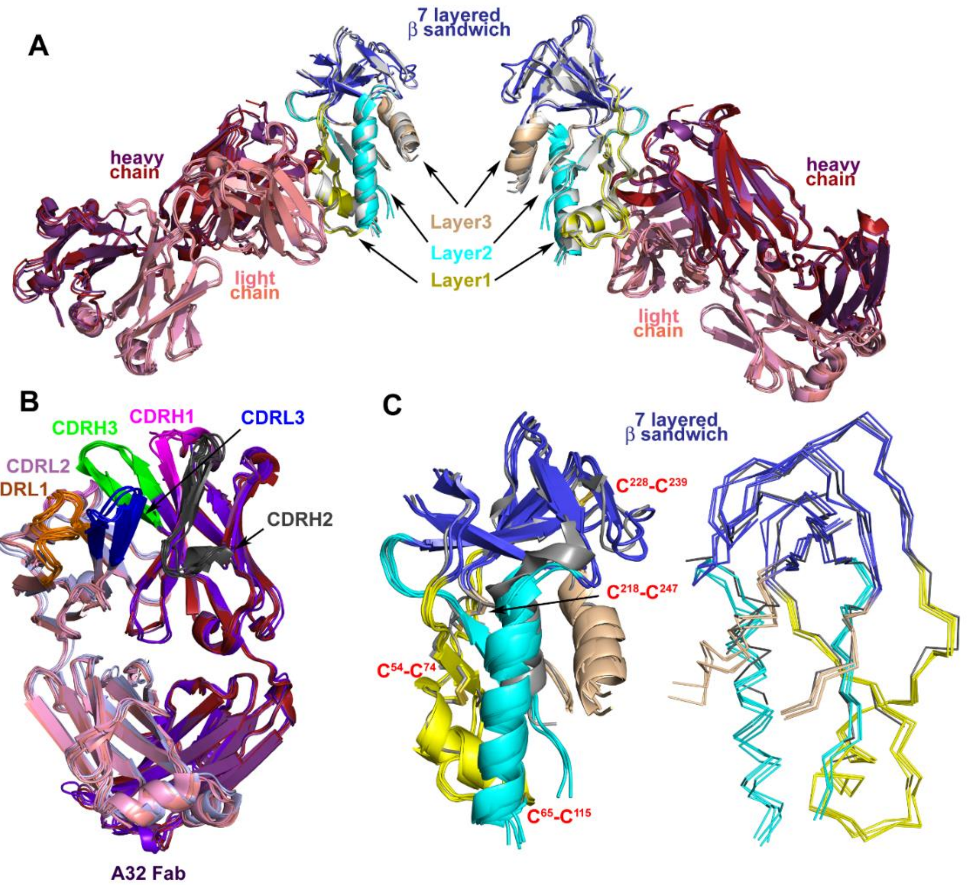

Figure S3, related to Figure 4. Structural comparison of A32 Fab-ID2 complexes (**A**) Superimposition of Fab A32-ID2 complex present in asymmetric unit of Fab A32-ID2$_{293\ HEK\ cells}$ and Fab A32-ID2$_{E.coli}$. ID2$_{E.coli}$ is colored yellow (layer 1), cyan (layer 2), wheat (layer 3), and blue (7-stranded β-sheet). ID2$_{293\ HEK\ cells}$ is colored grey. A32 Fab heavy and light chains are dark red and light red (*E. coli*) and purple and pink (293 HEK cells) respectively. (**B**) Superimposition of all the A32 Fabs from both crystal forms as well as the apo A32 Fab. The A32 Fab conformation changed little upon complex formation with an average RMSD between A32 Fab coordinates of 1.04 Å^2; the RMSD between two copies of the A32 Fab crystallized alone is 1.01 Å^2. (**C**) Superimposition of all four ID2's colored with layer coloring as above and the one ID1 is in grey. The 7-stranded β-sandwich region is well defined with a main chain RMSD between ID1 and ID2 in this region of 0.69 Å^2. Layer 3 loop residues in both ID1 and ID2 are disordered. The main chain RMSD between ID1 and ID2 is 1.36 Å^2 and the RMSD between ID2 and gp120 is 0.76 Å^2 as compared to the average RMSD between different copies of ID2 in both crystal forms of 0.67 Å^2.

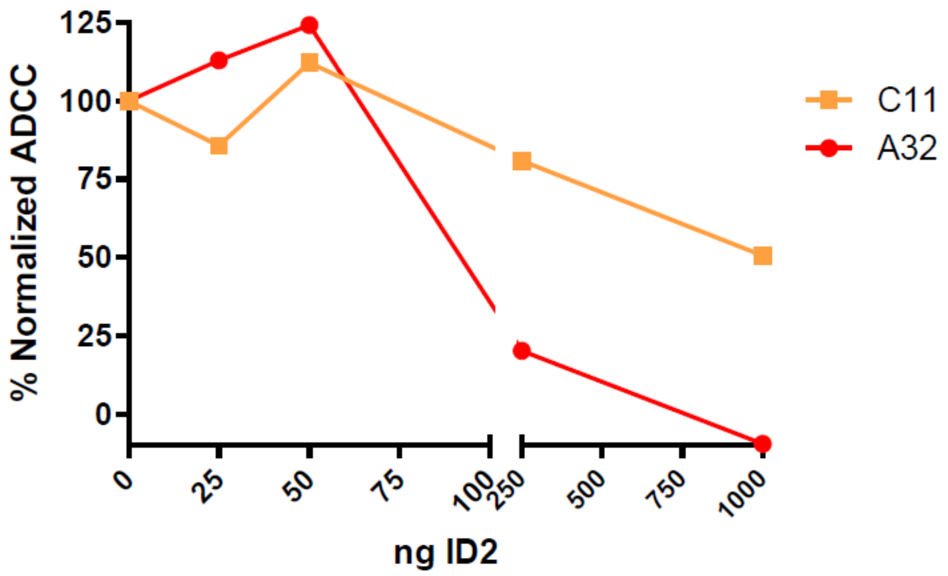

Figure S4, related to Figure 6A. ID-2 competition of ADCC activity mediated by A32 and C11 mAbs. CEM.NKr cells infected with Nef-Vpu- VSV-G pseudotyped NL4.3 GFP ADA were used at 48h post-infection for FACS-based ADCC assay using either A32 or C11 mAbs (500 ng) pre-incubated with indicated amounts of ID-2 protein for 30 min at room temperature. Data shown is the average of two different experiments

Supplementary References

Abacioglu, Y.H., Fouts, T.R., Laman, J.D., Claassen, E., Pincus, S.H., Moore, J.P., Roby, C.A., Kamin-Lewis, R., and Lewis, G.K. (1994). Epitope mapping and topology of baculovirus-expressed HIV-1 gp160 determined with a panel of murine monoclonal antibodies. AIDS Res Hum Retroviruses *10*, 371-381.

Bonsignori, M., Pollara, J., Moody, M.A., Alpert, M.D., Chen, X., Hwang, K.K., Gilbert, P.B., Huang, Y., Gurley, T.C., Kozink, D.M., *et al.* (2012). Antibody-dependent cellular cytotoxicity-mediating antibodies from an HIV-1 vaccine efficacy trial target multiple epitopes and preferentially use the VH1 gene family. J Virol *86*, 11521-11532.

Fouts, T.R., Tuskan, R., Godfrey, K., Reitz, M., Hone, D., Lewis, G.K., and DeVico, A.L. (2000). Expression and characterization of a single-chain polypeptide analogue of the human immunodeficiency virus type 1 gp120-CD4 receptor complex. J Virol *74*, 11427-11436.

Gohain, N., Tolbert, W.D., Acharya, P., Yu, L., Liu, T., Zhao, P., Orlandi, C., Visciano, M.L., Kamin-Lewis, R., Sajadi, M.M., *et al.* (2015). Cocrystal Structures of Antibody N60-i3 and Antibody JR4 in Complex with gp120 Define More Cluster A Epitopes Involved in Effective Antibody-Dependent Effector Function against HIV-1. J Virol *89*, 8840-8854.
